# Design and analysis of a fiber-optic sensing system for shape reconstruction of a minimally invasive surgical needle

**DOI:** 10.1038/s41598-021-88117-7

**Published:** 2021-04-21

**Authors:** Aizhan Issatayeva, Aida Amantayeva, Wilfried Blanc, Daniele Tosi, Carlo Molardi

**Affiliations:** 1 Department of Computer and Electrical Engineering, Nazarbayev University, Nur-Sultan, 010000 Kazakhstan; 2 Université Côte d’Azur, INPHYNI-CNRS UMR 7010, Nice, 06108 France; 3 National Laboratory of Astana, Laboratory of Biosensors and Bioinstruments, Nur-Sultan, 010000 Kazakhstan

**Keywords:** Optical sensors, Nanoparticles, Imaging and sensing, Biomedical engineering

## Abstract

This paper presents the performance analysis of the system for real-time reconstruction of the shape of the rigid medical needle used for minimally invasive surgeries. The system is based on four optical fibers glued along the needle at 90 degrees from each other to measure distributed strain along the needle from four different sides. The distributed measurement is achieved by the interrogator which detects the light scattered from each section of the fiber connected to it and calculates the strain exposed to the fiber from the spectral shift of that backscattered light. This working principle has a limitation of discriminating only a single fiber because of the overlap of backscattering light from several fibers. In order to use four sensing fibers, the Scattering-Level Multiplexing (SLMux) methodology is applied. SLMux is based on fibers with different scattering levels: standard single-mode fibers (SMF) and MgO-nanoparticles doped fibers with a 35–40 dB higher scattering power. Doped fibers are used as sensing fibers and SMFs are used to spatially separate one sensing fiber from another by selecting appropriate lengths of SMFs. The system with four fibers allows obtaining two pairs of opposite fibers used to reconstruct the needle shape along two perpendicular axes. The performance analysis is conducted by moving the needle tip from 0 to 1 cm by 0.1 cm to four main directions (corresponding to the locations of fibers) and to four intermediate directions (between neighboring fibers). The system accuracy for small bending (0.1–0.5 cm) is 90$\%$ and for large bending (0.6–1 cm) is approximately 92$\%$.

## Introduction

Minimally invasive surgery (MIS) is a type of surgery in which surgical instruments are inserted into the patient’s body through small incisions and the operation is conducted by a surgeon’s hand controlling the instruments from outside of the patient’s body^1^. The reliance of MIS on small incisions rather than large openings allows reducing the size and number of incisions in comparison with open surgeries, thus enabling less post-operative pain and faster recovery time for patients^2,3^. Most MIS procedures involves the insertion of the needles, minimally invasive applicators, endoscopes, and catheters inside the human body. The examples of such surgeries include biopsy, brachytherapy^4^, drug and anesthetic delivery (i.e. epidural anesthesia^5,6^), percutaneous robotics surgeries^7,8^, cancer treatment by thermal ablation^9^, endoscopic surgeries^10^ and others. The insertion of those surgical devices through the cavity of the body^11^ is complicated by the tortuosity of the paths^12^ and blindness of the procedure^13^, while the successful surgery requires precise positioning and movement of the instrument. Therefore, there is a need for an accurate navigation system for the correct positioning of the MIS devices.

Currently existing techniques used for positioning and navigation of the surgical devices inside the patient’s body can be divided into image-guided and electromagnetic (EM) tracking-based methods. Image-guided techniques include fluoroscopy, computer tomography (CT), magnetic resonance imaging (MRI), and ultrasound. Fluoroscopy^12,13^ and CT^14^ give satisfactory image-guidance during MIS, however they are accompanied by the exposure of the physicians and patients to ionizing radiation^12,15^. MRI does not emit radiation and provides real-time high contrast imaging modality^7^, but it operates in strong magnetic and radio-frequency fields and has a low refresh rate^16^. Ultrasound is an economically viable option, but it produces limited accuracy images due to low resolution and high signal to noise ratio^12^ adding small benefits to the MIS procedure. EM tracking-based system consists of a field generator creating the signal, several EM sensors detecting the signal, and a control unit, which creates a 3-dimensional map based on the change of each sensors’ positions^3,17^. The system gives accurate visual guidance and does not require a field of view, however, the tracking system is affected by EM distortions. Undesired EM fields, as well as interference from metal or ferromagnetic sources around the system, can result in a diminishing of measurement accuracy^3^.

Another promising method for MIS guidance is shape sensing of the needle based on the fiber optic sensors. Optical fiber characteristics, like biocompatibility, small size, and low mass^18^ make them suitable for integration with invasive surgical tools. Since the working principle of fibers is based on the optical signals^18^, they are not prone to EM fields and are compatible with MRI environment^19^. Moreover, fibers have real-time monitoring capability and the possibility of multiplexing of several sensors simultaneously along the same fiber^20,21^. The possibility of using fibers for shape sensing has appeared with the development of Fiber Bragg Grating (FBG). FBG is built in the way that the fiber core refractive index changes periodically that enables the selection of certain wavelength regions^22^. In other words, the narrow part of the wavelength spectrum named Bragg wavelength is rejected in transmission and transmitted in reflection. In case of physical parameter change such as temperature or strain variation, the interrogator detects Bragg wavelength shift^22^. FBG arrays, which is the incorporation of several FBGs in one fiber, allows wavelength division multiplexing. Battisti et al. propose the usage of the combination of FBG sensors and MRI for shape detection during brachytherapy^20^. Three fibers with 9 FBGs were put inside the stylet and inside the needle lumen^20^. The authors state that the accuracy of the deflection is 0.27 mm and needle position accuracy is 0.79 mm. Moreover, Battisti et al.^20^ mention that in comparison with MRI guidance the rate of the update was higher by 100 ms and the latency was lower by 300 ms.

The other fiber optic-based technology enabling shape sensing is the multi-core fiber Bragg grating (MCFBG) sensor^23^. Multi-core fiber (MCF) consists of multiple cores within the same cladding. Widespread MCF configurations are triangular with a central core and three outer cores, square and hexagonal ones^24^. The shape sensing accuracy improves when the number of cores increases while core to core distance does not change^24^. Several FBGs are inscribed in MCF forming MCFBG sensor. Khan et al. used triangular MCFBGs for the detection of 3D shape sensing of the catheter and endoscope^23^. The shape for MCFBG sensors is reconstructed with the calculation of individual strain of each core, then the curvature and torsion of each core are obtained from these strains, after that the central catheter curvature and torsion are calculated from each core curvature and torsion with a help of Frenet-Serrett equation^23,25^. The authors checked the setup in various configurations and achieved a maximum absolute error of 1.05 mm^23^. Fabrication of the MCFBG is more costly than single-mode fibers (SMF)^23^ and these fibers are influenced by the twisting effect that gives rise to uncertainty in bending direction parameters. Floris et al. suggested re-twisting and compensation of this twisting effect for MCFBG^26^.

FBG and MCFBG sensors, which are based on point to point sensing enabled by having multiple sensors along the fiber, have centimeter-scale spatial resolution^27,28^, which can be insufficient for small medical devices, like needles. The other sensing configuration that can have a better spatial resolution (close to millimeter-scale) is distributed sensing^29^. Distributed sensing that is configured with optical backscattering reflectometer (OBR) makes the whole fiber region sensitive to external changes, for example, strain alterations. OBR applies optical frequency domain reflectometry to demodulate spectrally reflected distributed signals. The reflection of the signals at each point happens due to Rayleigh Backscattering naturally occurring in the fiber^6^. However, even though OBR has a good spatial resolution, the interrogation with OBR is limited to only one fiber. Parent et al. have achieved multiplexing of the OBR by using the optical switch^30^. The authors have reconstructed the shape of a medical needle based on the system of three sensing fibers, which have been located 120 degrees from each other and placed inside the needle. The use of the optical switch limits the maximum acquisition rate to 0.5 Hz, and the positioning of the fibers inside the needle may create an obstacle for the flow of liquid (in case of using the needle for drug delivery)^30^.

The system discussed in this paper is also based on the OBR interrogation to exploit the high spatial resolution that it provides, but the multiplexing is achieved with Scattering Level Multiplexing (SLMux) methodology. SLMux is based on the use of two different types of fibers, such as SMFs and high-scattering MgO-nanoparticles doped fibers (NPDFs), produced by doping the cores of SMFs with MgO-based nanoparticles^31,32^. The Rayleigh backscattering level of NPDFs is 35–40 dB higher than the one of SMFs, which gives an opportunity to discriminate the two fibers. Four NPDFs have been spliced with SMF pigtails with different lengths in such a way that the SMF backscattering regions spatially separate four NPDFs from each other. In comparison with Parent et al.^30^ who applied three fibers and placed them inside the needle, in this setup four sensing fibers are glued outside of the needle at 90 degrees from each other. This fiber arrangement does not create an obstacle for the anesthetic and allows creating two pairs of opposite fibers (left and right, upper and lower). The distributed strain identified by the pair of left-right fibers is used to reconstruct the needle shape along the X-axis, while the strain detected by the upper-lower pair is converted to the needle shape along the Y-axis. Thus, the coordinate plane is created and the needle movement to all directions can be identified. Moreover, this arrangement of fibers can be useful to compensate for the influence of the temperature change on the measurements. Since the fibers are sensitive to both strain and temperature change, the difference between the temperature of the human body and the environment during the real MIS procedure can affect the strain measurement of the fibers. The two fibers located 180 degrees from each other detect almost the same strain with opposite signs, and almost the same temperature change. Therefore, the methodology to compensate for the temperature change has been also developed based on two pairs of opposite fibers and will be presented in another paper. The results of the preliminary experiments with the insertion of this needle into a custom-made phantom have shown a good potential of the suggested technology^6,33^. The needle is a Tuohy needle used for epidural anesthesia and the custom-made phantom mimics the spinal anatomy of the human body, so the system has been suggested for epidural anesthesia. However, it can be used with some modifications to guide the rigid surgical instruments for other MIS procedures.

In this paper, the performance analysis and accuracy estimation of the developed system is presented. The performance of the shape reconstruction is evaluated by fixing the base of the needle while moving its tip with respect to the base from 0 to 1 cm with a step of 0.1 cm to four main directions (the sides of the needle with fibers) and to four intermediate directions (between each pair of neighboring fibers). The reconstructed needle shape is compared with the reference and the reconstruction error for each direction is estimated. The accuracy of the needle shape reconstruction is evaluated separately for small bending of 0.1–0.5 cm and for large bending of 0.6-1 cm.

## Results

### Raw strain detected by the interrogation method

**Figure 1:**
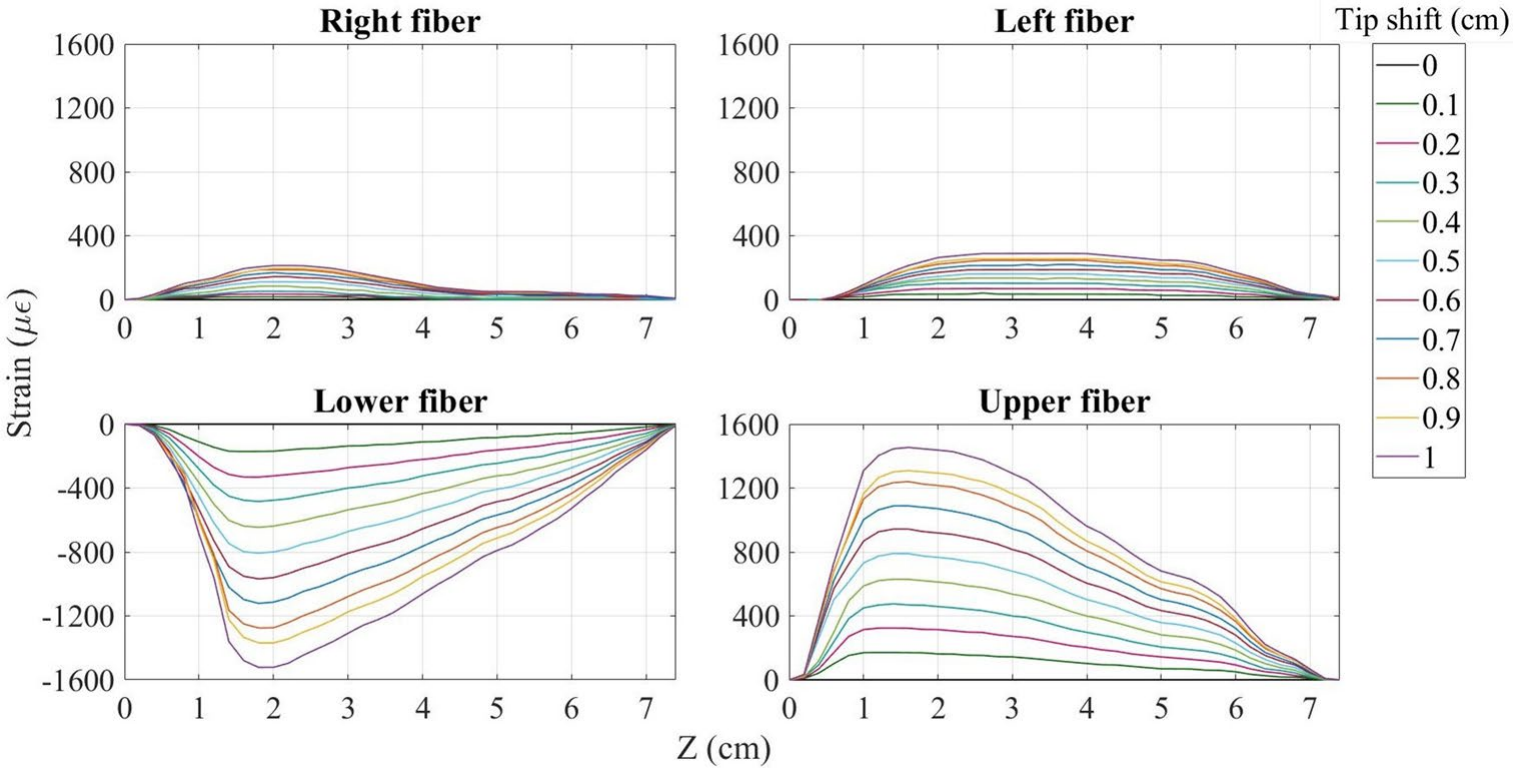
Strain detected by all four fibers over the length of the needle during its bending to the lower direction.

The interrogator used in this experiment is able to detect the strain sensed by each of the fibers connected to it (see the “Methods” section for more details). Figure 1 illustrates the strain detected by each fiber during the needle inclination to the lower direction (along the Y-axis). Since the lower side of the needle experiences compression and the opposite side stretches, the strain detected by the lower fiber is negative, while the upper fiber senses almost the same but positive strain. In general, the pattern for the upper-lower fibers is that for the tip shift by 0.1 cm the maximum strain is detected by the points located 1.5–2 cm from the base of the needle and constitutes about 150 $\upmu \upepsilon$. With each step of the tip shift, the strain increases by approximately 150 $\upmu \upepsilon$ reaching 1500 $\upmu \upepsilon$ at the end. The needle displacement causes the right and left fibers also experience bending because they are inclined together with the needle. Therefore, as can be seen in Fig. 1, right and left fibers also feel some strain. However, the strain detected by the right-left pair is different from the strain pattern of upper-lower fibers: first of all, it is much smaller (less than 300 $\upmu \upepsilon$) and secondly, the two opposite fibers experience the strain with the same sign. Therefore, the direction of the needle bending can be identified based on the pair of fibers, which experience opposite strain.

Strain sensed by the fibers during the needle bending to all other directions are given in Supplementary Fig. S1. According to this figure, when the needle is bent to the upper direction, the elongated lower fiber detects the positive strain and the compressed upper fiber feels the negative strain. During the needle bending to the left and right (along the X-axis), the pair of left-right fibers becomes active and experiences similar strain with opposite signs depending again on the direction of movement, while the upper-lower pair detects small strain with the same sign. The needle inclination to the intermediate directions is characterized by all four fibers being active and sensing the strain of about 1000 $\upmu \upepsilon$ for the maximum tip movement. For the case of intermediate directions, both pairs of fibers detect the strain with opposite sign, which implies that the bending has been conducted along both axes (X and Y). The results show that the strain detected by each fiber highly depends on the direction of the needle bending, and thus can be used for the identification of the needle shape. This gives an opportunity to develop an algorithm which reconstructs the needle shape based on the strain detected by two pairs of opposite fibers. The algorithm itself is discussed in “Methods” section and the result of the application of this algorithm is presented below.

### Needle shape reconstruction

**Figure 2:**
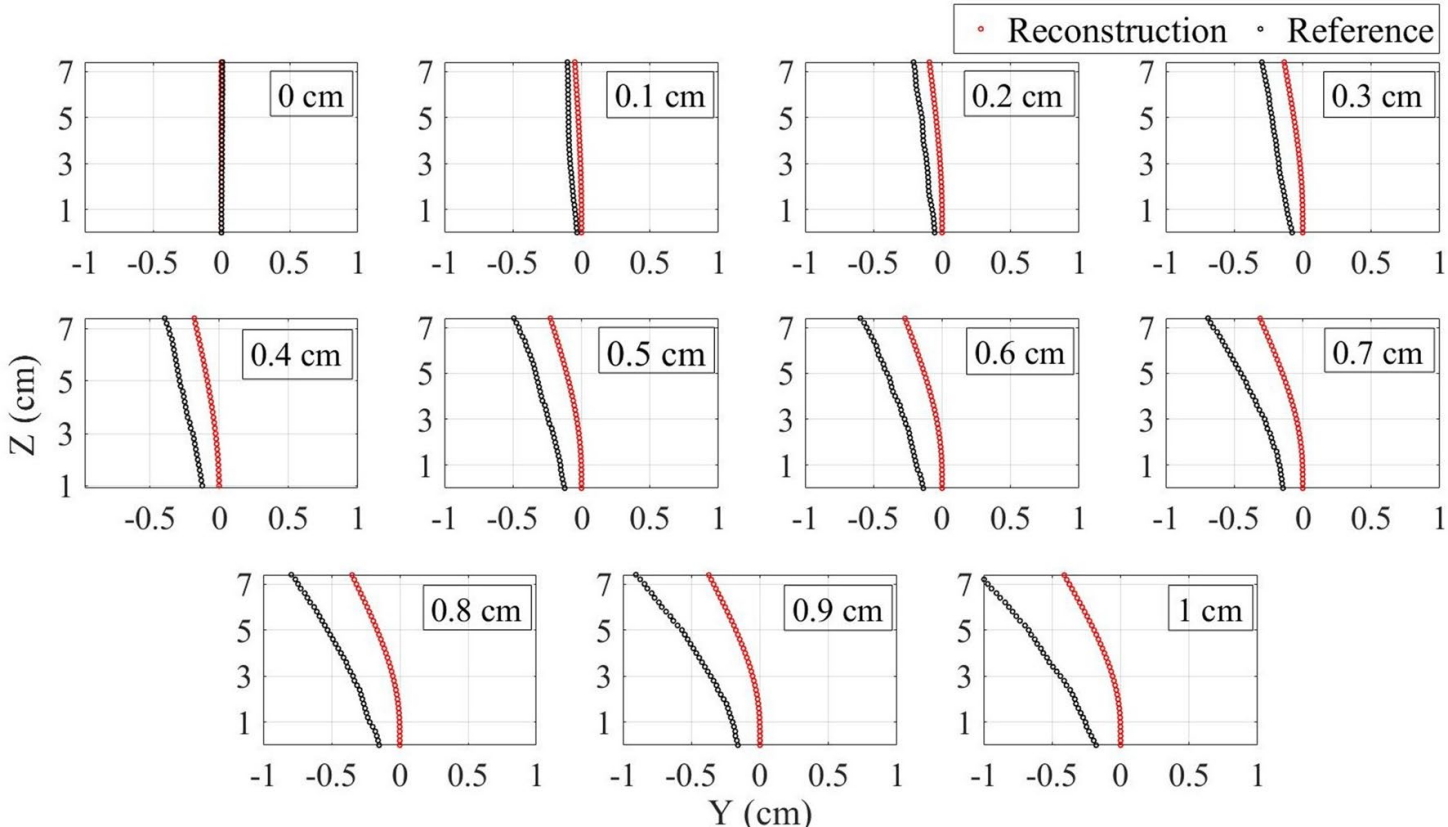
Comparison of the reconstructed shape (red) with the reference (black) before the application of the correction coefficients during the needle bending to the lower direction.

**Figure 3:**
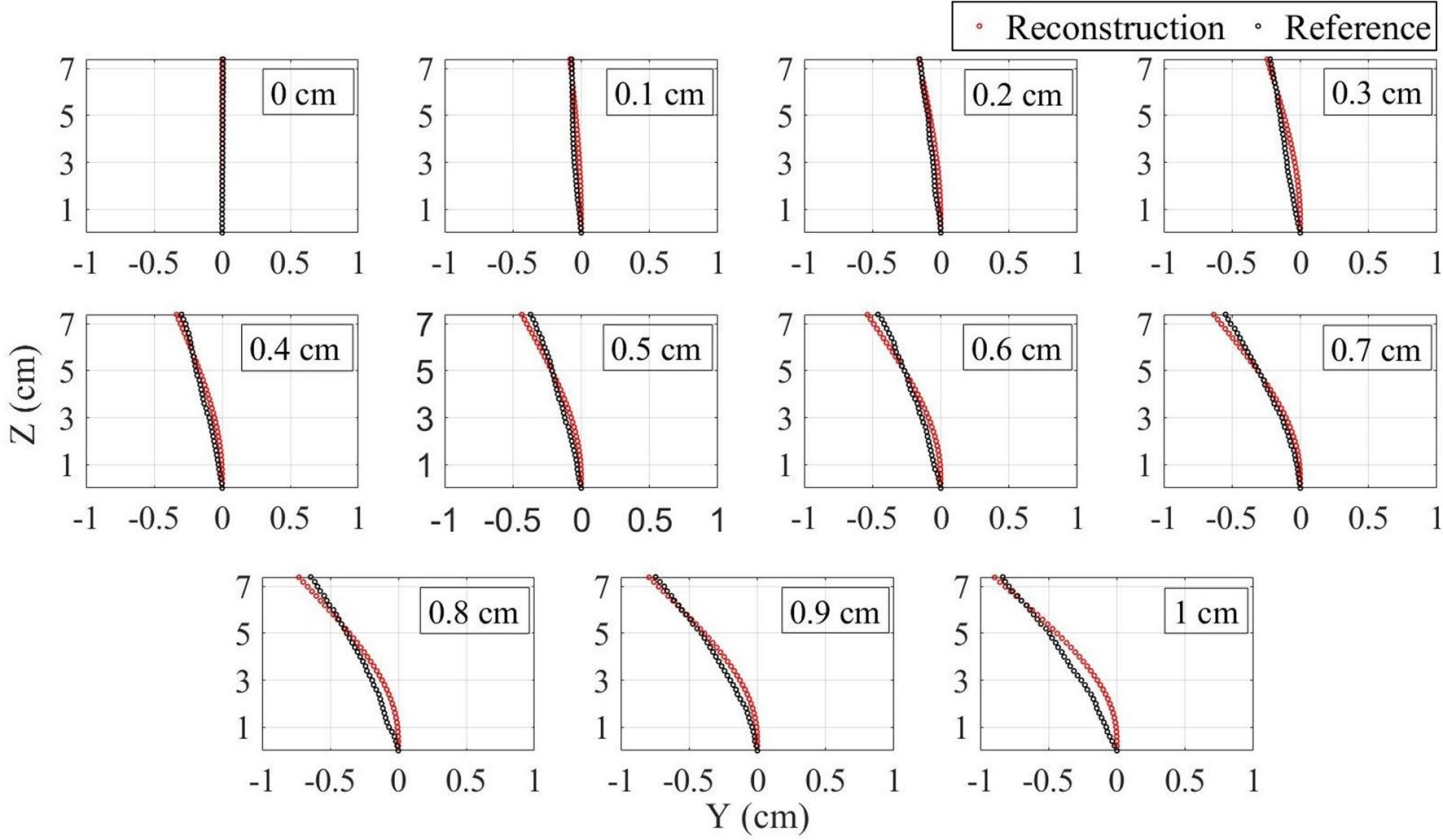
Comparison of the reconstructed shape (red) with the reference (black) after the application of the correction coefficients during the needle bending to the lower direction.

The strain data is used for the shape reconstruction algorithm, which is discussed in detail in the “Methods” section. Figure 2 presents the needle shape reconstructed with the use of this algorithm (red) compared with the reference (black) during the needle inclination to the lower direction. First of all, the base of the referenced shape (1st sensing point) shifts from 0 cm along the Y-axis as the tip moves. That is because, as it can be noticed in Fig. 7e, the fibers right near to the needle base are not glued firmly, so the strain measurement starts at about 0.6 cm from the base. Therefore, that first sensing point shifts with respect to the base of the needle when it is bent. Since the aim of the algorithm is to find the relative needle bending, the reference has been moved so that its first sensing point is at the origin and the reconstructed shape has been compared with the modified reference. Secondly, the reconstructed shape is underestimated although the direction of the inclination has been identified correctly. The possible reason of such underestimation is that the glue limits the fibers’ flexibility which leads to the detection of only a partial strain . In order to compensate for this influence, the correction coefficients have been calculated for both pairs of fibers (see the “Methods” section). The correction coefficient for the right–left pair of fibers found to be equal to 1.39295 $\times$ x + 1.91441, where x is the x coordinate of the sensing points along the fibers. The correction coefficient for the pair of upper-lower fibers is 1.25813 $\times$ y + 1.54276, where y is the y coordinate of the sensing points. Figure 3 illustrates the comparison of the needle shape after the application of correction coefficient (red) with the reference moved to the origin (black). As can be seen, the algorithm is able to reconstruct the shape with quite a good precision.

**Figure 4:**
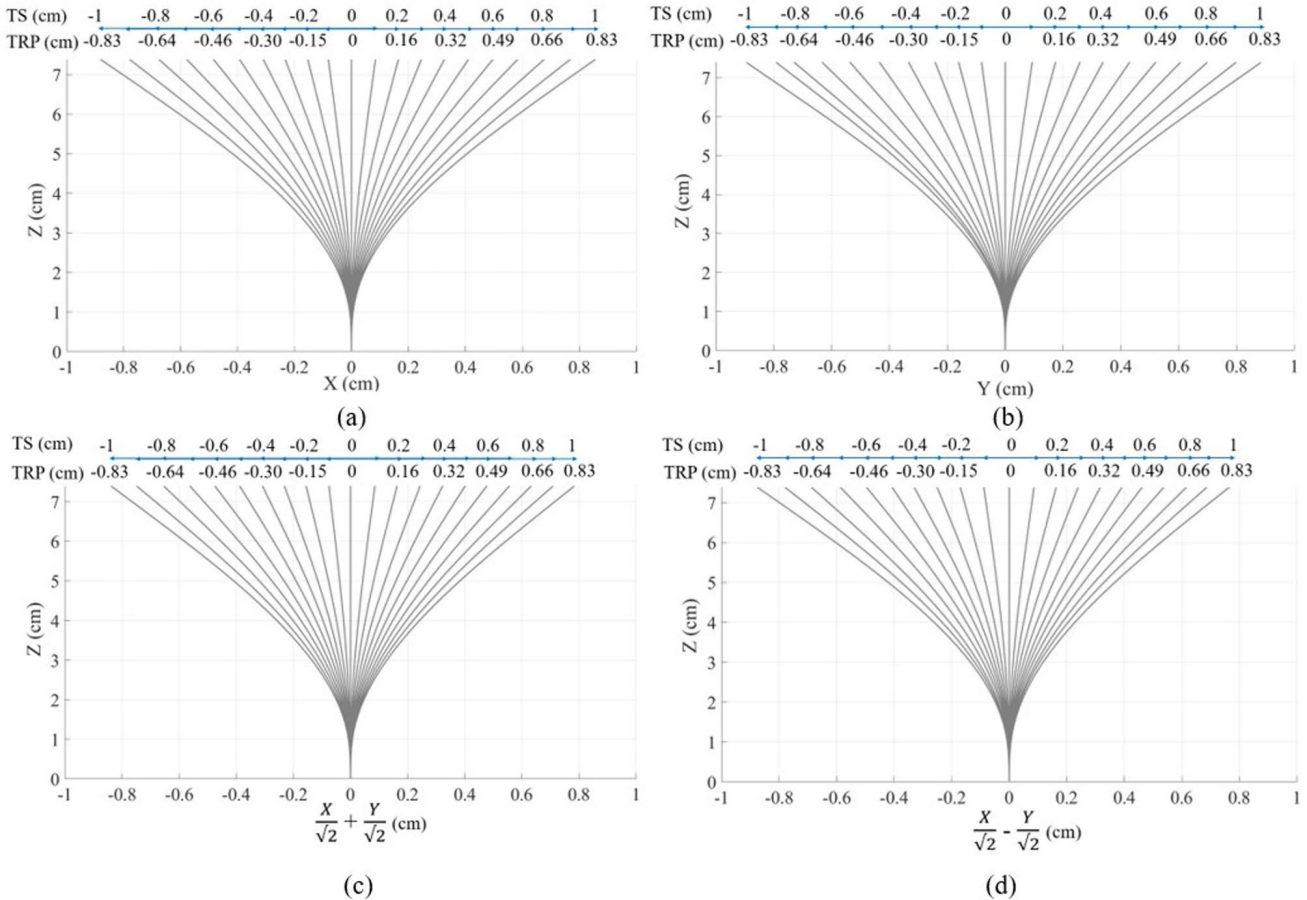
Needle shape reconstruction during the tip movement from 0 to 1 cm with a step of 0.1 cm for all directions: (**a**) left and right; (**b**) upper and lower; (**c**) up-right and low-left; (**d**) low-right and up-left. In this figure, TS is the tip shift relative to the needle base and TRP is the tip relative position with respect to the first sensing point.

The comparison of the reconstructed shape before and after the application of the correction coefficients with the reference for all other bending directions is shown in Supplementary Figs. S2 and S3. It can be seen, that the reconstruction algorithm has succeeded in restoration of the needle shape during its bending to all eight directions (even to intermediate). Figure 4 illustrates the reconstructed shape separately from the reference for all directions combining the left and right (a), upper and lower (b), up-right and low-left (c), low-right and up-left (d) directions. The blue axis in Fig. 4 shows the tip shift according to the reference. The numbers shown above the blue axis represents the tip displacement from 0 to 1 cm with a step of 0.1 cm with respect to the base of the needle. However, as discussed above, the reconstruction of the needle has been conducted relative to its first sensing point (0.6 cm from the base), therefore the numbers below the blue axis show the tip position relative to the first sensing point. The reconstructed shapes of the needle are in agreement with the tip relative position found from the reference. For instance, the maximum tip shift detected by the algorithm for all directions is about 0.8–0.85 cm which corresponds to the tip relative position of 0.83 cm.

## Discussion

**Figure 5:**
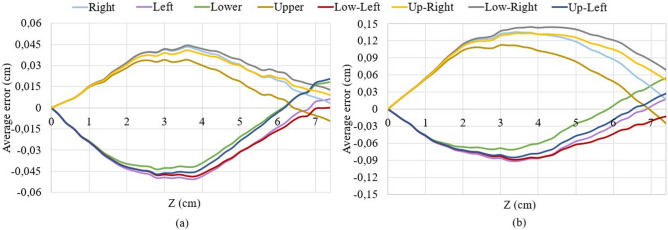
Reconstruction errors for all directions for the small bending of 0.1–0.5 cm (**a**) and large bending of 0.6–1 cm (**b**).

According to Fig. 4, the algorithm after application of correction coefficient has succeeded in the reconstruction of the shape in both main and intermediate directions. However, when comparing the reconstructed shape with the reference (Fig. 3) it can be seen that there is still a small difference in the red and black shapes. This difference is the reconstruction error of the algorithm that has been shown in Fig. 5 separately for each direction for small (0.1–0.5 cm) and large (0.6–1 cm) bending. The average error patterns for all bending directions are quite similar to each other, but the right, up-right and low-right directions differs a little from the rest. First of all, the tip position for the three mentioned directions is underestimated, while it is overestimated for the other five directions. Secondly, the average errors for the right, up-right, and low-right directions are higher than for the rest of the directions for larger bending. There are several possible reasons which resulted in such difference:

1. *Fibers gluing technique* the fibers have been glued along the needle manually and although they were aligned as precise as possible, there is still a possibility of small misalignment.
2. *The influence of glue itself* it can differ a little from fiber to fiber and even from one part of the fiber to another part of the same fiber.
3. *Reconstruction of the reference* the reference has been reconstructed by taking photos of each position of the needle and processing them with the online program. The program allows to find the approximate coordinates of the sensing points along the needle and, thus, reconstruct its shape. The use of this program can also introduce some small mismatches.

According to Fig. 5, the maximum average error of the algorithm after the application of correction coefficient corresponds to the middle of the needle (3–4.5 cm) and this is true for the bending to all eight directions. For the small bending the maximum average error is about 0.045 cm (Fig. 5a), while for the large bending the maximum average error is in the range 0.06–0.15 cm (Fig. 5b).

**Figure 6:**
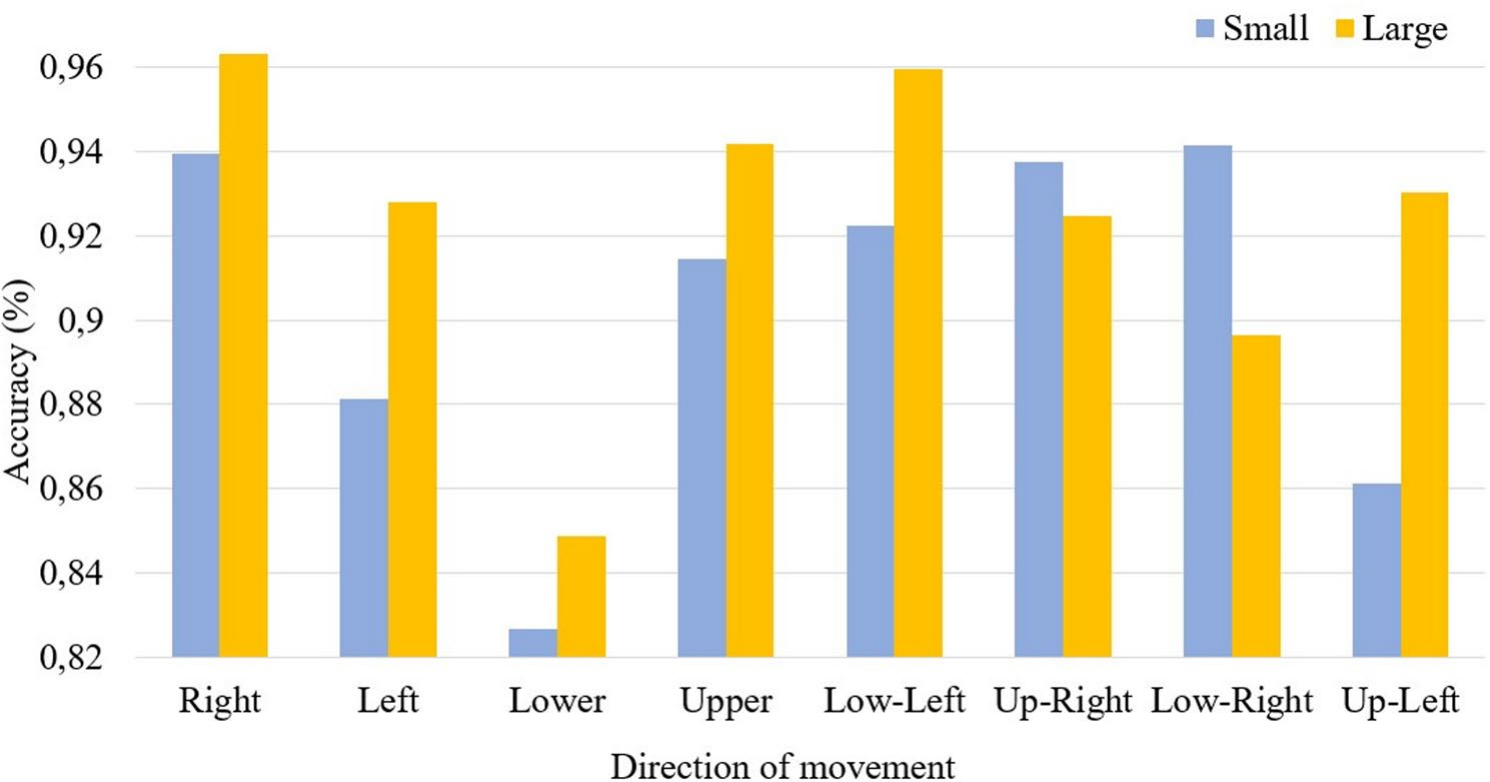
Accuracy of the needle tip position identification for small bending of 0.1–0.5 cm and large bending of 0.6–1 cm.

During the minimally invasive surgeries, the aim is to position the needle tip correctly. Therefore it is important that the maximum reconstruction error of the proposed system corresponds to the middle of the needle, not its tip. According to Fig. 5, the needle tip position has been underestimated during its movement to the right, up-right, low-right, and low left directions by a maximum of 0.013 cm for the small bending and 0.07 cm for large bending. For the other four directions, the needle tip position has been overestimated by a maximum of 0.02 cm for small bending and 0.06 cm for large bending. Figure 6 illustrates the accuracy of the tip position estimation which is calculated as the reconstructed tip position over the reference. As can be seen, the accuracy ranges from 82 to 97%, and it is generally higher for the large bending than for the small bending. The average accuracy for all directions for the small bending is 90$\%$ and for the large bending is 92$\%$.

In order to illustrate the performance of the system for the reconstruction of the needle behavior during its real-time movement, the visualization experiment has been conducted. The needle has been moved so that its tip draws different shapes (square, plus sign, multiplication sign, and sand clock) and that has been recorded on the video. The videos that show the top view of the real movement of the needle and the reconstructed behavior of the needle are given in the Supplementary information (Videos S1–S4). As can be seen, despite the small imperfections of the setup, the system can be used for the real-time visualization of the needle behavior with an average accuracy of 90–92$\%$.

## Methods

**Figure 7:**
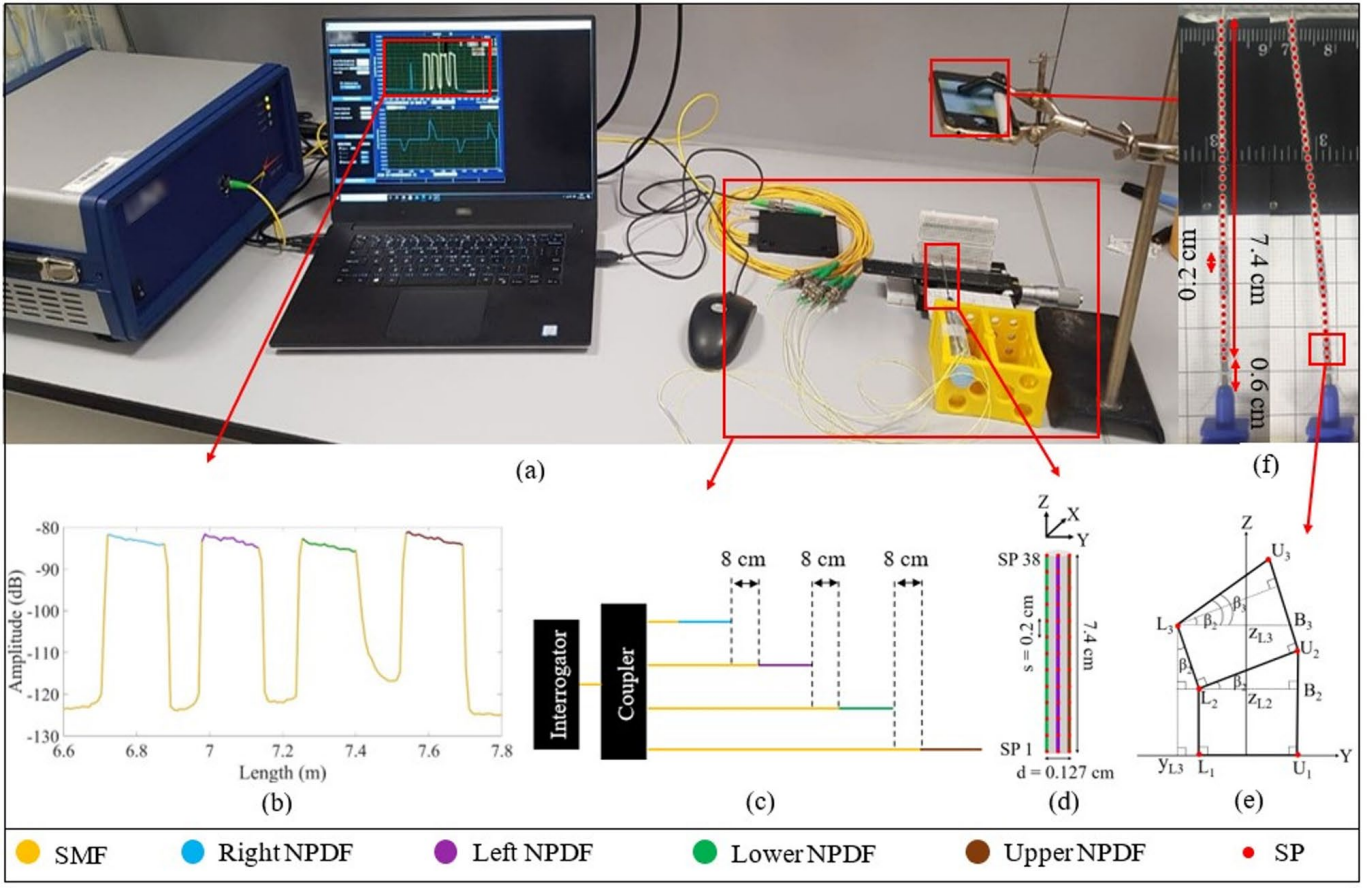
Experimental setup: (**a**) The photo of the setup consisting of the Luna OBR interrogator with the evaluation software, 1 by 4 coupler connecting the interrogator output to the four SMFs of different lengths spliced with NPDFs, the epidural needle equipped with four NPDFs and held by the yellow fixation box, the micropositioner which moves the tip of the needle from 0 to 1 cm with a 0.1 cm step, the phone which takes photos of the needle position in order to reconstruct the real shape of the needle using the online program; (**b**) backscattering spectrum of four NPDFs spliced with SMFs detected by the interrogator; (**c**) the schematics of the multiplexing of the NPDFs; (**d**) The schematics of the NPDFs arrangement on the needle; (**e**) The schematics of needle during bending to the lower direction (first three SP levels); (**f**) examples of the needle positions photos after the processing with the online program. The program gives the coordinates of the sensing points (red dots) along the needle.

Figure 7 shows the experimental setup used to conduct the performance analysis of the needle shape reconstruction algorithm.

The shape reconstruction is based on the four optical fibers glued around the needle and the Luna OBR interrogator (Luna OBR 4600, Luna Inc., USA). In this section the working principle and multiplexing of the Luna OBR, the needle shape reconstruction algorithm and the procedure of its performance analysis will be discussed.

### The OBR interrogator: working principle and multiplexing method

The working principle of the Luna OBR is based on the Rayleigh backscattering which is the scattering of light from each section of the fiber^30^. In this experiment, the length of the sections along the fiber is set to 0.5 cm. The instrument detects the spectrum of the backscattered light from each section and produces the plot of spectrum over length of the fiber (as shown in Fig. 7b). External physical factors that influence the fiber, like the application of the temperature or strain, causes the spectral shift of the backscattered light. The spectral shift is calculated by correlating the backscattering spectrum of each section of the fiber with the spectrum of the same section of the fiber recorded before it has been exposed to the temperature or strain. The temperature or strain are proportional to the spectral shift and can be found by the coefficients of − 0.8014 $^{\circ }$C/GHz and − 6.6680 $\upmu \upepsilon$/GHz respectively^30^. In this experiment, the temperature is constant, while the strain changes due to the needle bending.

The main reason for using the Luna interrogator is to achieve distributed strain measurement along all four fibers. The standard SMFs are not appropriate for multiplexing because they have the same scattering level and their backscattered spectra overlap. Therefore, high-scattering NPDFs have been produced by doping MgO nanoparticles to the core of SMFs (the fabrication process is discussed in the papers^31,32^). The scattering power of NPDFs is 35–40 dB higher than the scattering level of SMFs, which allows achieving the Scattering-Level Multiplexing (SLMux). Four NPDFs of size 15 cm are spliced with the SMF pigtails in such a way that each SMF is about 8 cm longer than the preceding SMF + NPDF fiber (see Fig. 7c). Four SMF+NPDF fibers have been connected to the interrogator through a 1 by 4 coupler. The NPDFs are used as the sensing fibers, and each sensor can be discriminated from others because of the 8 cm lower-scattering SMF regions between them. Figure 7b illustrates the spectrum of the SLMux setup, which consists of four high-scattering (about − 85 dB) regions of NPDFs separated by low-scattering (− 125 dB) SMF regions. Strain measurements, that have been shown in Fig. 1, are taken from the spectral shift of that four regions of high-scattering fibers.

### Fibers gluing technique

**Figure 8:**
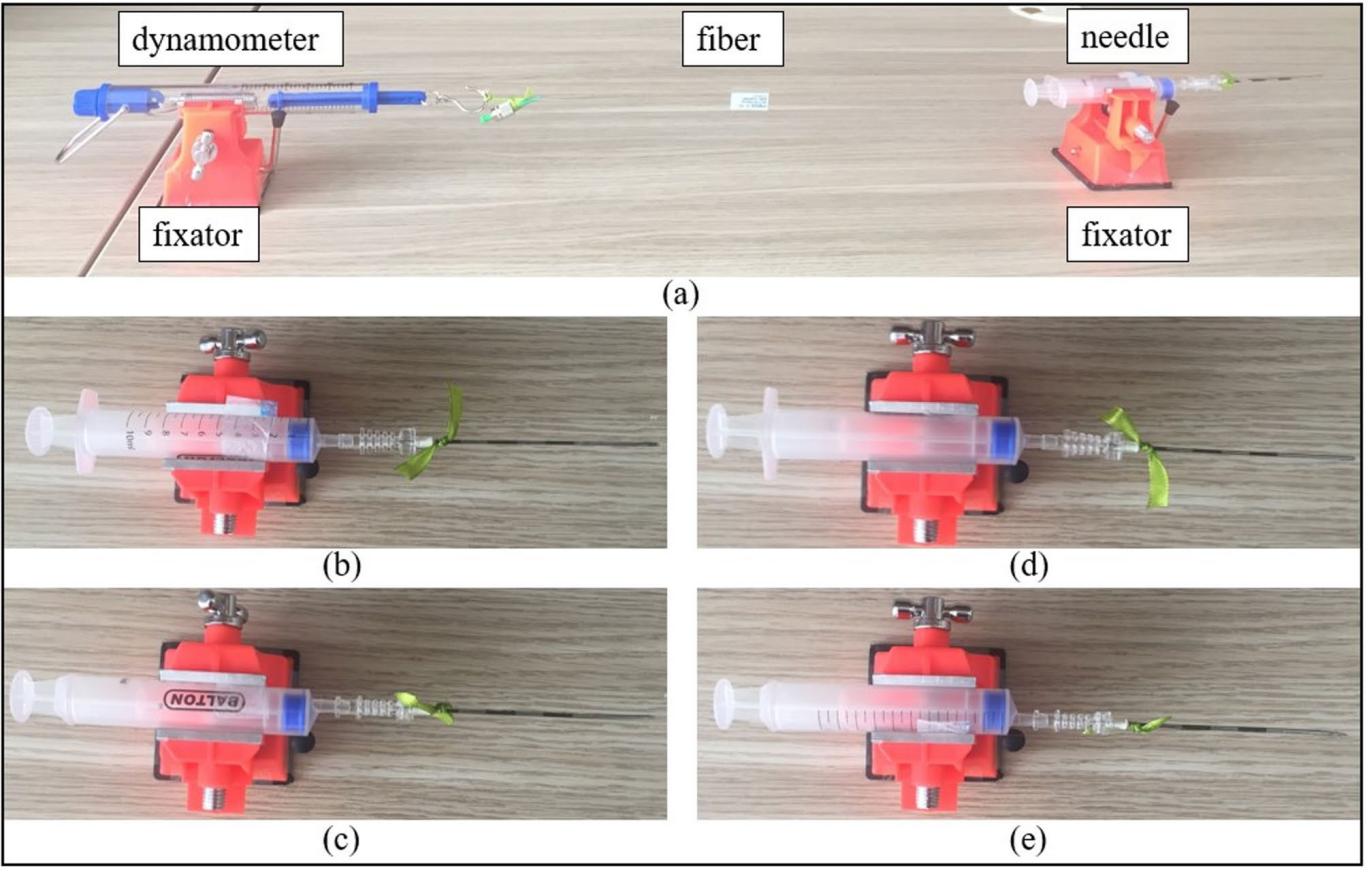
Setup for pasting optical fibers to the needle (**a**) which is performed by placing the needle on the fixator, pasting the fiber on the needle tip, and stretching the fiber by the dynamometer to glue the rest of the fiber. The needle has been rotated by 90 degrees to paste upper (**b**), right (**c**), lower (**d**), and left (**e**) fibers.

As can be seen in Fig. 7d, four NPDFs are glued along the needle (d = 0.127 cm) at 90 degrees from each other. The attaching of the fibers to the needle has been performed manually and the setup consists of the needle, four fibers, the dynamometer, and two fixators (see Fig. 8a)

The procedure of gluing the fibers to the needle:

1. About 1 cm of the first fiber is pasted to the tip of the needle by epoxy glue and is left for half a day in order for the glue to dry up.
2. After the tip of the fiber is firmly fixed, the needle is placed into the fixator in such a way that the fiber is on the upper side of the needle. The fixator is attached to the table with double-sided scotch tape. The fiber is stretched to make it straight by the dynamometer up to the point when it shows 100 g (to achieve consistent tension for all four fibers). The dynamometer is fixed by another fixator. About 5 cm of the fiber is pasted to the needle with the epoxy glue and left for half a day to dry up.
3. The fiber near the needle base (about 1.5 cm) is left unglued because it is not firmly fixed to the needle due to the dynamometer stretching. Therefore, the remained part of the fiber is fixed with a thread and pasted by the epoxy glue for another half a day.
4. After pasting the first fiber, the needle is rotated by 90 degrees from the position (b) to (c) shown in Fig. 8, and the procedure is repeated for the right side of the needle. Then the needle is rotated by another 90 degrees (Fig. 8d) and the fiber is pasted on the lower side of the needle. Finally, the needle is positioned as in Fig. 8e and the lower fiber is glued.

This manual gluing technique has been used for this experiment to check the feasibility of the needle shape reconstruction algorithm. However, it is planned to improve the gluing technique in order to achieve more accurate fibers’ positioning along the needle.

### Shape reconstruction: fibers arrangement and reconstruction algorithm

The arrangement of four fibers at 90 degrees from each other allows to achieve two pairs of the opposite fibers (left-right and upper-lower) and use each pair to reconstruct the needle shape along X and Y axes respectively. The algorithm for the needle shape reconstruction along the Y-axis, presented in Fig. 7e, consists of the following steps:

1. The distributed sensing provided by the Luna OBR allows obtaining sensing points (SP) located each s = 0.2 cm along the lower and upper fibers. In this experiment, the monitored length of the needle is 7.4 cm, so there are 38 SPs along each fiber. The sensor spacing and number of SPs may be increased depending on the needle dimensions, but for this needle, the 0.2 cm resolution has been found to be sufficient to reconstruct the shape accurately.
2. Each fiber is divided by the SPs into 37 sections. The elongation or compression of each section is calculated by assuming that the strain sensed by the section is the average of the strains detected by the two neighboring SPs ($\epsilon _{L_{i}}$ and $\epsilon _{L_{i+1}}$) and multiplying this strain by the length of the section before the strain has been applied (s). Thus, the length of the elongated or compressed $i_{th}$ sections of the lower ($S_{L_{i}}$) and upper ($S_{L_{i}}$) fibers are calculated as follows:
  1 $$\begin{aligned} S_{L_{i}}= & {} s + s\times \frac{\epsilon _{L_{i}} + \epsilon _{L_{i+1}}}{2}, \end{aligned}$$
  2 $$\begin{aligned} S_{U_{i}}= & {} s+ s\times \frac{\epsilon _{U_{i}} + \epsilon _{U_{i+1}}}{2}. \end{aligned}$$
3. After that the coordinates of the ending points of the first sections of the lower and upper fibers are calculated as follows:
  3 $$\begin{aligned} y_{L_{2}}= & {} -\frac{d}{2}, \end{aligned}$$
  4 $$\begin{aligned} y_{U_{2}}= & {} \frac{d}{2}, \end{aligned}$$
  5 $$\begin{aligned} z_{L_{2}}= & {} S_{L_{2}}, \end{aligned}$$
  6 $$\begin{aligned} z_{U_{2}}= & {} S_{U_{2}}. \end{aligned}$$
4. When the needle is bent to the lower direction, the lower fiber sections experience compression, while the opposite upper fiber is elongated, and vice versa. This results in the difference between the lengths of the two opposite sections which is used to calculate the bending angle of the subsequent section of the needle. The bending angle ($\beta _{i}$) is the angle between the line which connects two opposite SPs ($L_{i}U_{i}$) and the Y-axis. As can be seen in Fig. 7e, the first bending angle is 0 and the second bending angle can be calculated as follows:
  7 $$\begin{aligned} \beta _{2} = \arctan \left( \frac{U_{2}B_{2}}{d}\right) = \arctan \left( \frac{S_{U_{1}}-S_{L_{1}}}{d}\right) , \end{aligned}$$
5. As it can be seen in Fig. 7e, the angle $\angle L_{3}L_{2}H$ = 90 - $\beta _{2}$, so the angle $\angle L_{2}L_{3}H$ = $\beta _{2}$. Therefore, the coordinates of the subsequent sections’ end can be found from the sections length known from step 2 and the coordinates of the previous SPs:
  8 $$\begin{aligned} y_{L_{3}}= & {} y_{L_{2}} + S_{L_{2}}\times sin(\beta _{2}), \end{aligned}$$
  9 $$\begin{aligned} y_{U_{3}}= & {} y_{U_{2}} + S_{U_{2}}\times sin(\beta _{2}), \end{aligned}$$
  10 $$\begin{aligned} z_{L_{3}}= & {} z_{L_{2}} + S_{L_{2}}\times cos(\beta _{2}), \end{aligned}$$
  11 $$\begin{aligned} z_{U_{3}}= & {} z_{U_{2}} + S_{U_{2}}\times cos(\beta _{2}). \end{aligned}$$
6. The bending angle of the subsequent section is calculated as a summation of the previous bending angle with the angle between the line connecting the two opposite beginning SPs ($L_{2}U_{2}$) and the line connecting the two corresponding ending SPs ($L_{3}U_{3}$):
  12 $$\begin{aligned} \beta _{3} = \beta _{2}+ \arctan \left( \frac{S_{U_{2}}-S_{L_{2}}}{d}\right) . \end{aligned}$$
7. This process continues, until the whole needle inclination to the upper or lower directions is reconstructed.
8. Upper and lower fibers are used to reconstruct the shape of the needle along the Y-axis, so in order to detect the needle movement along the X-axis the same algorithm is applied for the pair of left–right fibers. After that, the coordinates of the points along the middle of the needle are calculated: x coordinates are the average of x coordinates of the left and right fibers’ sensing points, y coordinates are the average of y coordinates of the upper-lower fibers’ points and z coordinates are the average of the z coordinates of all four fibers’ points. To reconstruct the whole needle in 3 dimensions, the points located $\frac{d}{2}$ cm from the middle are drawn. Figures 2, 3 and 4 show the movement of the middle of the needle, and Supplementary Videos S1–S4 illustrates the 3D bending of the needle.

### Performance analysis: experiment conduction and data processing

In order to evaluate the performance of the needle shape reconstruction system described above, the needle calibration experiment has been conducted. The base of the needle has been positioned inside the yellow box (see Fig. 7a) so that the base is firmly fixed but the needle itself can move freely. The tip of the needle has been fixed with the breadboard, which has been glued to the micropositioner. This setup allows moving the needle tip from 0 to 1 cm with a step of 0.1 cm. The needle has been moved to the left and right directions (along X-axis), then rotated by 90 degrees and moved to the upper and lower directions (along Y-axis). Moreover, the needle has also been moved to the intermediate directions, such as upper-right and lower-left (along $\frac{X}{\sqrt{2}} + \frac{Y}{\sqrt{2}}$ axis), upper-left and lower-right (along $\frac{X}{\sqrt{2}} - \frac{Y}{\sqrt{2}}$ axis). A mobile phone camera, aligned with the plane of the needle, has been used to take the photos of each position of the needle. The photos have been processed with the “Web Plot Digitizer” online program^34^, which helps to find the coordinates of the points along the needle and based on them reconstruct its shape. Figure 7f illustrates the two positions of needle which moved from 0 to 1 cm. The red dots along the needle drawn by the mentioned online program, are located approximately in the middle between the SPs of the left and right fibers. The coordinates of those points are used to approximately reconstruct the real shape of the needle during the experiment, which is used as a reference in the further analysis.

Five trials of the movement to each direction have been performed. The trials have shown consistent results, therefore in this paper, only one of the trials for each direction has been presented. The reconstructed shape has been compared with the reference obtained using the online program (see Fig. 2). In our algorithm, the aim is to find the relative positions of all the needle with respect to the starting point so the reference shapes have been all moved so that the starting point is at 0 cm. The reconstructed shape has been then compared with the modified reference and the coefficient that corrects the reconstructed shape has been found for each trial of the experiment. The coefficients from all trials of the movement to the left and right have been averaged and found to be 1.39295 $\times$ x + 1.91441, where x is the x coordinate of the points along the needle. The same has been done for all trials of the movement to the upper and lower directions and the coefficient for that pair has been found to be 1.25813 $\times$ y + 1.54276. The coefficients have compensated for the influence of the glue which resulted in only partial detection of the needle bending by fibers. The algorithm has been modified with the consideration of the two coefficients for left–right and upper-lower pairs of fibers and the shape has been reconstructed again (see Fig. 3). The same coefficients have been used to reconstruct the shape when the needle has been moved to all directions, including intermediate ones (see Supplementary Fig. S2). In order to better illustrate the needle movement by steps along four axes (main and intermediate) the reconstructed shapes for the needle bending to the pairs of opposite directions have been combined and presented separately from the reference in Fig. 4.

The errors for the small (0.1–0.5 cm) and large (0.6–1 cm) bending shown in Fig. 5a,b respectively) have been estimated by finding the average errors (the reference minus the reconstructed shape) of all five trials separately for each direction. The accuracy of the tip position shown in Fig. 6 has been calculated as an average of the accuracy (reconstructed tip position over the reference tip position) of all five trials separately for small and large bending.

## Conclusion

This paper has presented the system for shape reconstruction of the rigid medical needle based on distributed strain measurement achieved by gluing four optical fibers at four sides of the needle. The strain sensed by the pair of opposite fibers during the needle movement is able to reconstruct the needle inclination along one axis. Two pairs of the opposite fibers allow to create a coordinate plane and reconstruct the needle bending to any direction. The setup has been calibrated by the correction coefficient and its performance has been tested by moving the needle to four main directions (sides where fibers are located) and four intermediate directions (in between each two neighboring fibers). The system has shown promising results by allowing to identify the position of the tip with the accuracy of $90\%$ for small bending (0.1–0.5 cm) and $92\%$ for large bending (0.6–1 cm). The system is proposed to be used for minimally invasive surgeries to visualize the behavior of the needle during its insertion into the human body.

Future work will be aimed at the improvement of the setup, in particular, modifying the fibers gluing technique and finding the bio-compatible glue. The manual gluing of the fibers has introduced the aforementioned errors and the improved protocol of fiber gluing may improve the accuracy of the setup. One of the possible ways to achieve the precise straightforward gluing of each fiber and exactly 90 degrees distance between them is to produce four microgrooves along the needle and glue the fibers inside them. Moreover, the epoxy glue used to attach the fibers in this setup may not be suitable for the real medical surgeries, therefore it is important to find a bio-compatible alternative. After that, it is planned to conduct a set of experiments with the real medical phantom and use the shape reconstruction data to classify different types of insertion (wrong or correct). As a result, the system can be real-time guidance for minimally invasive surgeries, which can not only visualize the behavior of the needle but also notify about correctness or incorrectness of the insertion in real time.

## Supplementary information

Supplementary Information 1.

Supplementary Video S1.

Supplementary Video S2.

Supplementary Video S3.

Supplementary Video S4.

## References

[1] Kuo C, Dai JS (2009). Robotics for minimally invasive surgery: A historical review from the perspective of kinematics. Int. Symp. Hist. Mach. Mech..

[2] Vitiello V, Lee S-L, Cundy TP, Yang G-Z (2013). Minimally invasive surgery. IEEE Rev. Biomed. Eng..

[3] Sorriento A (2020). Optical and electromagnetic tracking systems for biomedical applications: A critical review on potentialities and limitations. IEEE Rev. Biomed. Eng..

[4] Leibinger A, Oldfield MJ, Rodriguez y Baena F (2016). Minimally disruptive needle insertion: A biologically inspired solution. Interface Focus.

[5] Ng KW, Goh JQ, Foo SL, Ting PH, Lee TK (2013). Needle insertion forces studies for optimal surgical modeling. Int. J. Biosci. Biochem. Bioinform..

[6] Beisenova A (2019). Distributed fiber optics 3D shape sensing by means of high scattering NP-doped fibers simultaneous spatial multiplexing. Opt. Express.

[7] Li G (2020). Body-mounted robotics for interventional MRI procedures. IEEE Trans. Med. Robot. Bionics.

[8] Pedram, S. A., Ferguson, P., Ma, J., Dutson, E. & Rosen, J. Autonomous suturing via surgical robot: An algorithm for optimal selection of needle diameter, shape, and path. In *Proc. -IEEE Int. Conf. on Robotics Autom.* 2391–2398 (2017).

[9] Elgezua I, Kobayashi Y, Fujie MG (2013). Survey on current state-of-the-art in needle insertion robots: Open challenges for application in real surgery. Procedia CIRP.

[10] Gao RY (2020). Overlooked risk for needle tract seeding following endoscopic ultrasound-guided minimally invasive tissue acquisition. World J. Gastroenterol..

[11] Tendick F, Sastry SS, Fearing RS, Cohn M (1998). Applications of micromechatronics in minimally invasive surgery. IEEE/ASME Trans. Mechatron..

[12] Shi C (2017). Shape sensing techniques for continuum robots in minimally invasive surgery: A survey. IEEE Trans. Biomed. Eng..

[13] Han Z (2019). A targeting method for robot-assisted percutaneous needle placement under fluoroscopy guidance. Comput. Assist. Surg..

[14] Zhang TF (2019). Lesion positioning method of a CT-guided surgical robotic system for minimally invasive percutaneous lung. Int. J. Med. Robot. Comput. Assist. Surg..

[15] Harrison Farber S (2020). Correction to: Radiation exposure to the surgeon during minimally invasive spine procedures is directly estimated by patient dose. Eur. Spine J..

[16] Wei J, Wang S, Li J, Zuo S (2017). Novel integrated helical design of single optic fiber for shape sensing of flexible robot. IEEE Sensors J..

[17] Song S, Li Z, Yu H, Ren H (2015). Electromagnetic positioning for tip tracking and shape sensing of flexible robots. IEEE Sensors J..

[18] Keeffe SO, Mccarthy D, Woulfe P, Grattan MWD, Hounsell AR (2015). A review of recent advances in optical fibre sensors for in vivo dosimetry during radiotherapy. Br. J. Radiol..

[19] Morana A (2017). Radiation-hardened fiber Bragg grating based sensors for harsh environments. IEEE Trans. Nucl. Sci..

[20] de Battisti MMMB, de Senneville JJWRD, Vulpen MV (2016). Fiber Bragg gratings-based sensing for real-time needle tracking during MR-guided brachytherapy. Med. Phys..

[21] Zheng D, Madrigal J, Chen H, Barrera D, Sales S (2017). Multicore fiber-Bragg-grating-based directional curvature sensor interrogated by a broadband source with a sinusoidal spectrum. Opt. Lett..

[22] Gusarov A, Hoeffgen SK (2013). Radiation effects on fiber gratings. IEEE Trans. Nucl. Sci..

[23] Khan F (2019). Multi-core optical fibers with Bragg gratings as shape sensor for flexible medical instruments. IEEE Sensors J..

[24] Lo Presti D (2020). Fiber Bragg gratings for medical applications and future challenges: A review. IEEE Access.

[25] Floris, I., Adam, J. M., Calderòn, P. A. & Sales, S. Measurement uncertainty of 7-core multicore fiber shape sensors. In *Proc. Seventh Eur. Work. on Opt. Fibre Sensors* 22 (2019).

[26] Floris I, Madrigal J, Sales S, Calderón PA, Adam JM (2020). Twisting measurement and compensation of optical shape sensor based on spun multicore fiber. Mech. Syst. Signal Process..

[27] Tosi D, Schena E, Molardi C, Korganbayev S (2018). Fiber optic sensors for sub-centimeter spatially resolved measurements: Review and biomedical applications. Opt. Fiber Technol..

[28] Lindner E (2014). Trends and future of fiber Bragg grating sensing technologies: Tailored draw tower gratings (DTGs). Opt. Sensing Detect. III.

[29] Sales S, Barrea D, Hervás J, Madrigal J (2019). Microwave photonics for optical fiber sensors. Opt. InfoBase Conf. Pap..

[30] Parent F (2017). Enhancement of accuracy in shape sensing of surgical needles using optical frequency domain reflectometry in optical fibers. Biomed. Opt. Express.

[31] Blanc W (2011). Fabrication of rare earth-doped transparent glass ceramic optical fibers by modified chemical vapor deposition. J. Am. Ceram. Soc..

[32] Blanc W, Dussardier B (2016). Formation and applications of nanoparticles in silica optical fibers. J. Opt..

[33] Beisenova A (2019). Simultaneous distributed sensing on multiple MgO-doped high scattering fibers by means of scattering-level multiplexing. J. Light. Technol..

[34] Rohatgi, A. *Web Plot Digitizer* (2020). https://automeris.io/WebPlotDigitizer/.

