# Supplementary material for: Design and analysis of a fiber-optic sensing system for shape reconstruction of a minimally invasive surgical needle
Source: Sci Rep. 2021 Apr 21;11:8609. doi: 10.1038/s41598-021-88117-7 (PMC8060330; doi:10.1038/s41598-021-88117-7)
Supplement: Supplementary file 1 — Supplementary Information 1. [file 41598_2021_88117_MOESM1_ESM.docx]

**Design and analysis of a fiber-optic sensing system for shape reconstruction of a minimally invasive surgical needle**

**Aizhan Issatayeva**^1^**, Aida Amantayeva**^1^**, Wilfried Blanc**^2^**, Daniele Tosi**^1,3^**, and Carlo Molardi**^1,*^

^1^Nazarbayev University, Department of Computer and Electrical Engineering, Nur-Sultan, 010000, Kazakhstan

^2^Universit ´e Cˆote d’Azur, INPHYNI–CNRS UMR 7010, Nice, 06108, France

^3^National Laboratory of Astana, Laboratory of Biosensors and Bioinstruments, Nur-Sultan, 010000, Kazakhstan

^*^Correspondence: carlo.molardi@nu.edu.kz

**Supplemetary Information**

Figure S1 shows the strain pattern for each fiber during needle bending to three main directions, namely upper (a), left (b), and right (c), and to four intermediate directions, including low-left (d), up-right (e), up-left (f), and low-right (g). As can be seen, during the needle inclination to the main directions (a, b, c) the fiber located on the side of the bending direction (upper, left, right for a, b, c respectively) expriences compression and negative strain, while the opposite fiber (lower, right, left for a, b, c respectively) stretches resulting in the positive strain. The pair of non-active fibers (right-left for a and upper-lower for b, c) detects relatively small strain with the same sign. This pattern corresponds to the one shown in Fig. 1, which presents the results for needle bending to the lower direction. During needle bending to the intermediate directions (d, e, f, g), the negative strain is detected by the two fibers between which the bending occurs (lower and left for d, upper and right for e, upper and left for f, lower and right for g), while the two opposite fibers experience similar but positive strain.


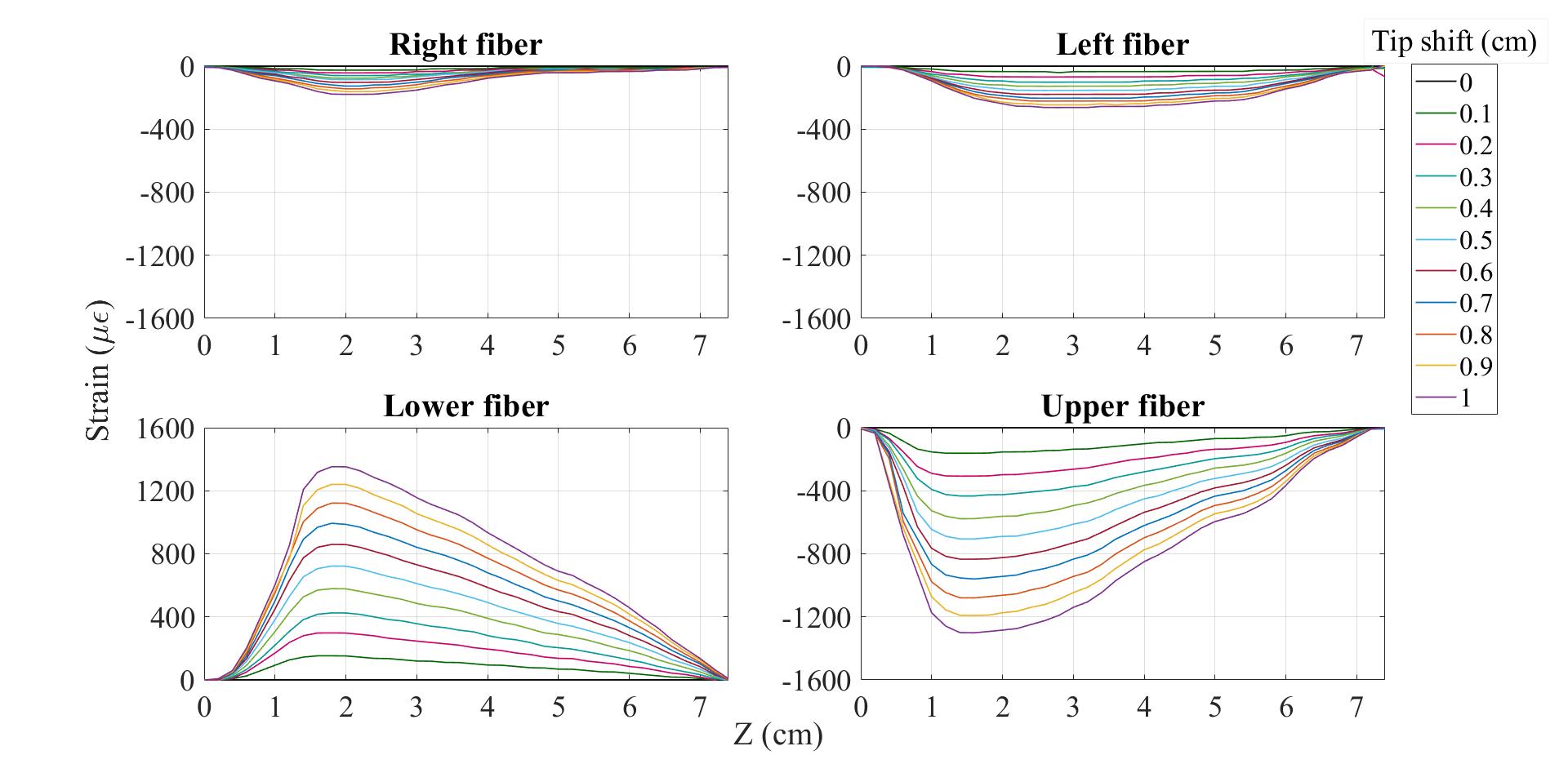


1. Needle bending to the upper direction


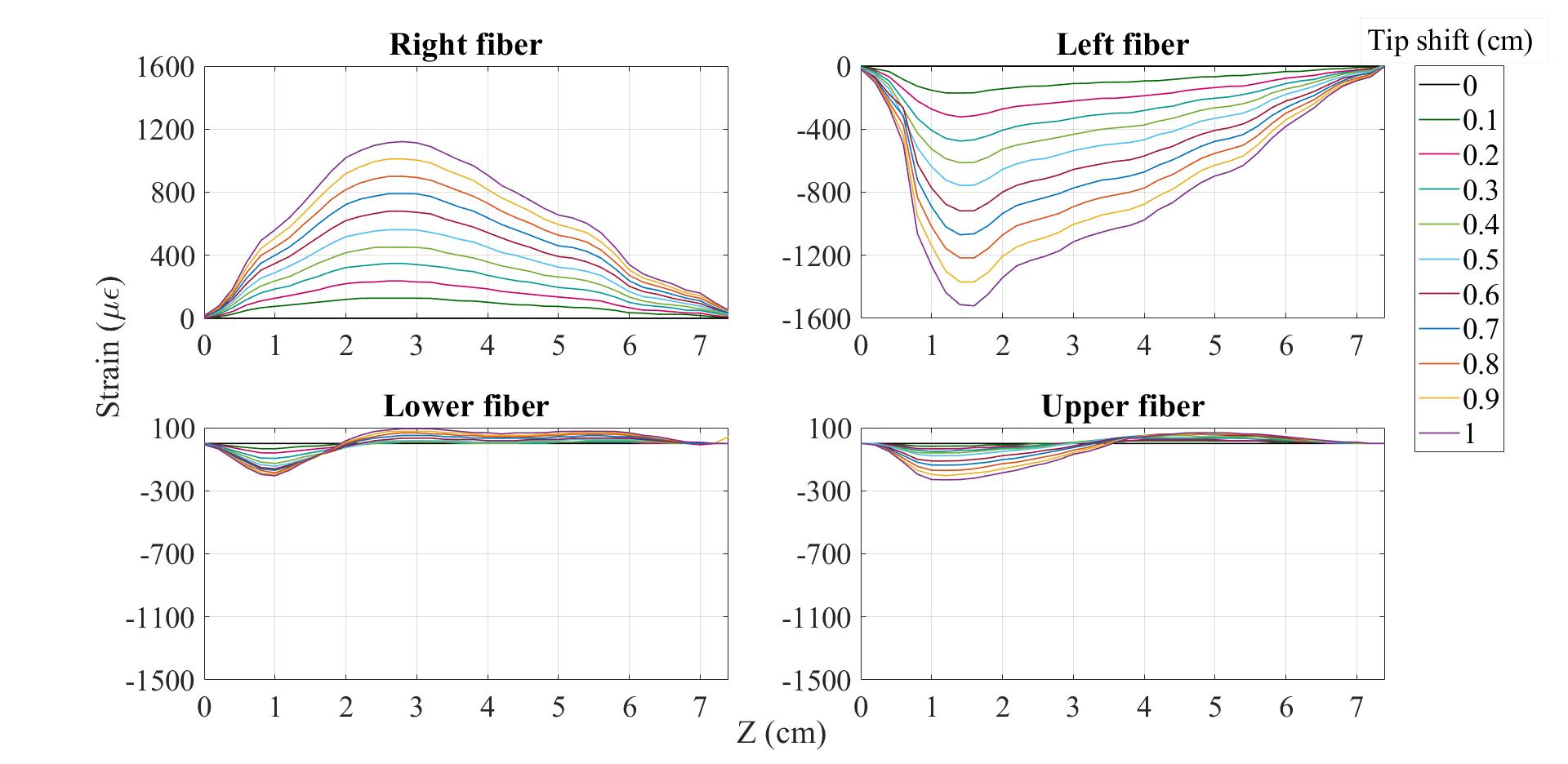


1. Needle bending to the left direction.


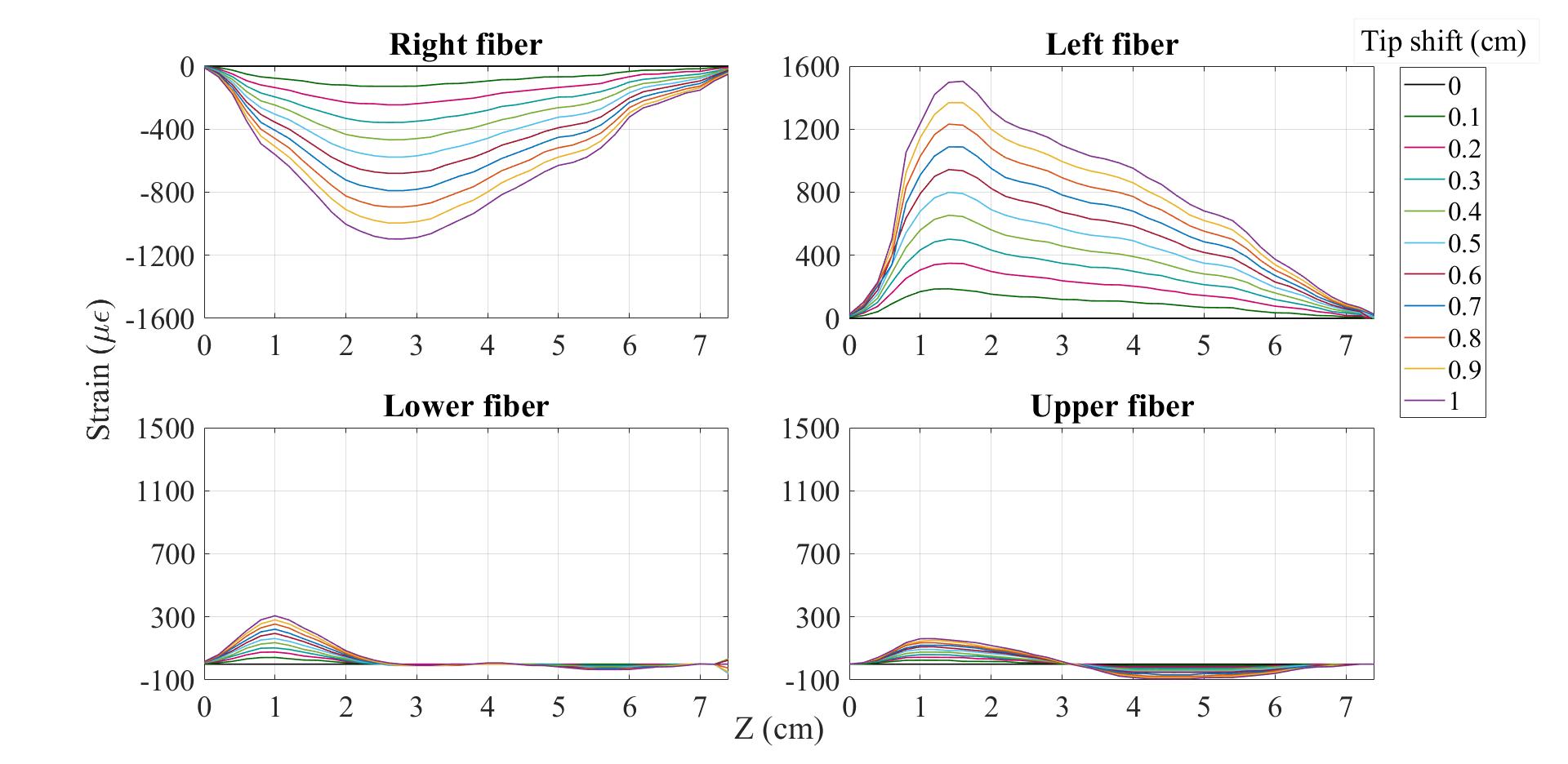


1. Needle bending to the right direction.


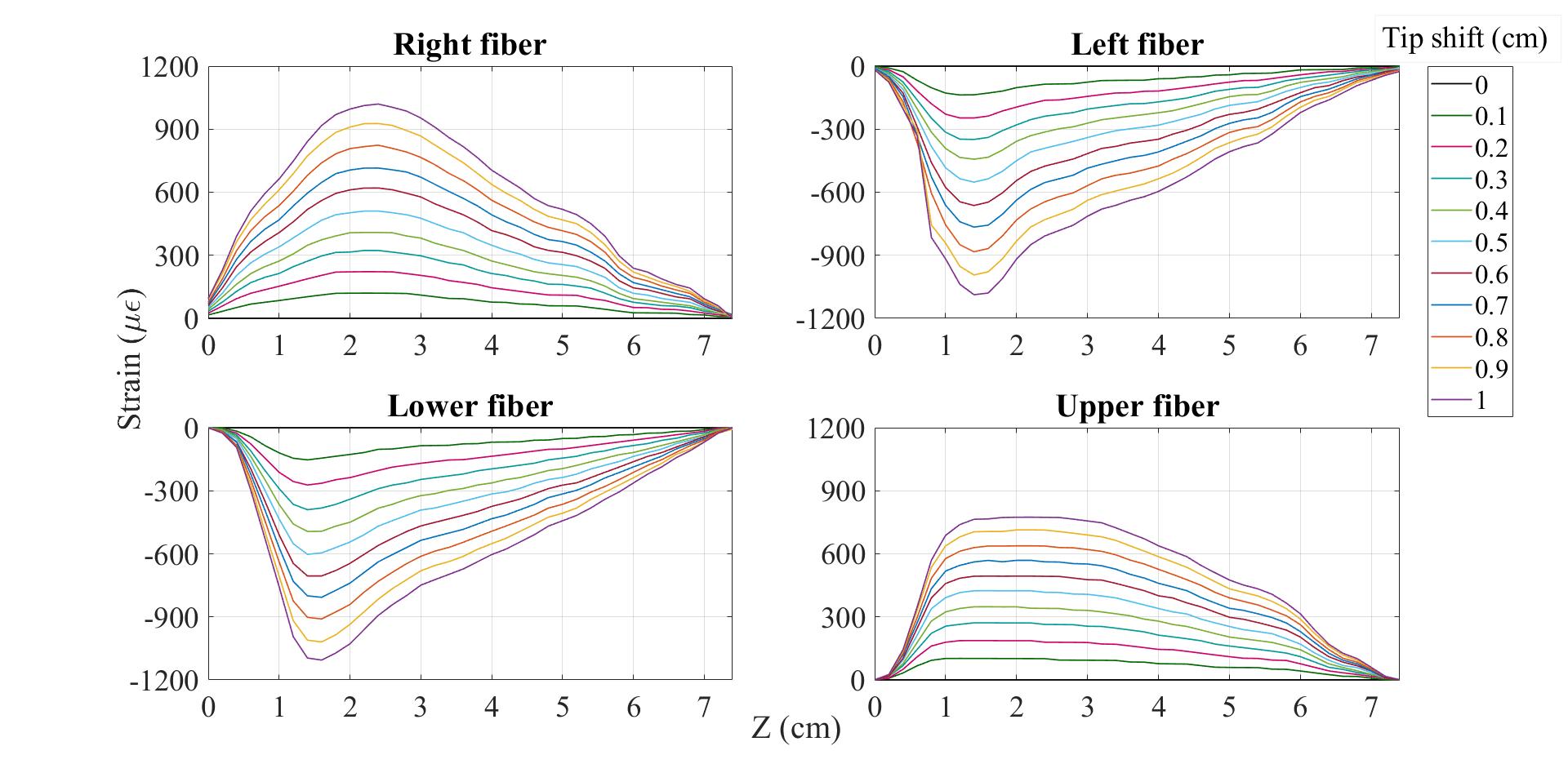


1. Needle bending to the low-left direction.


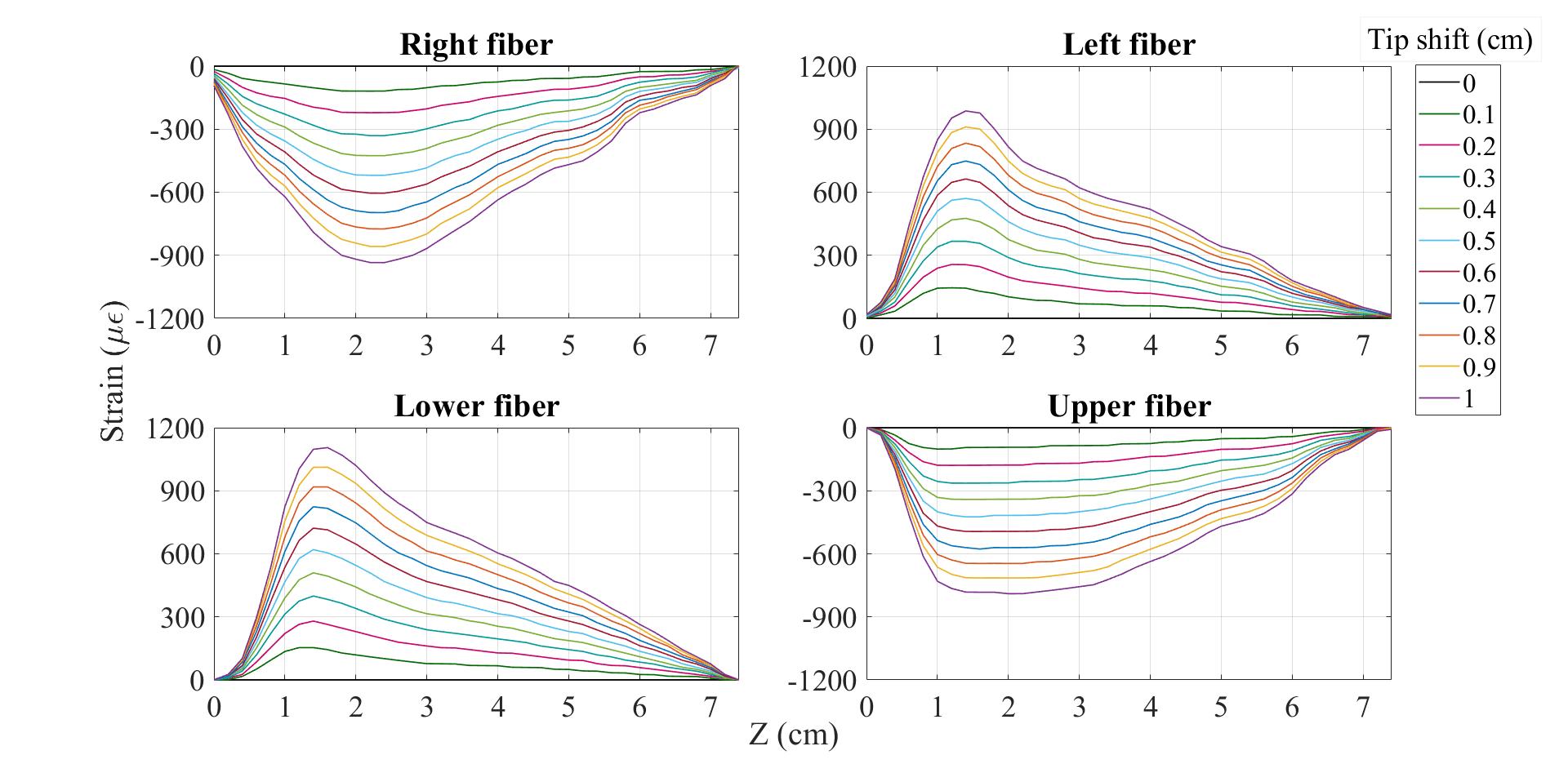


1. Needle bending to the up-right direction.


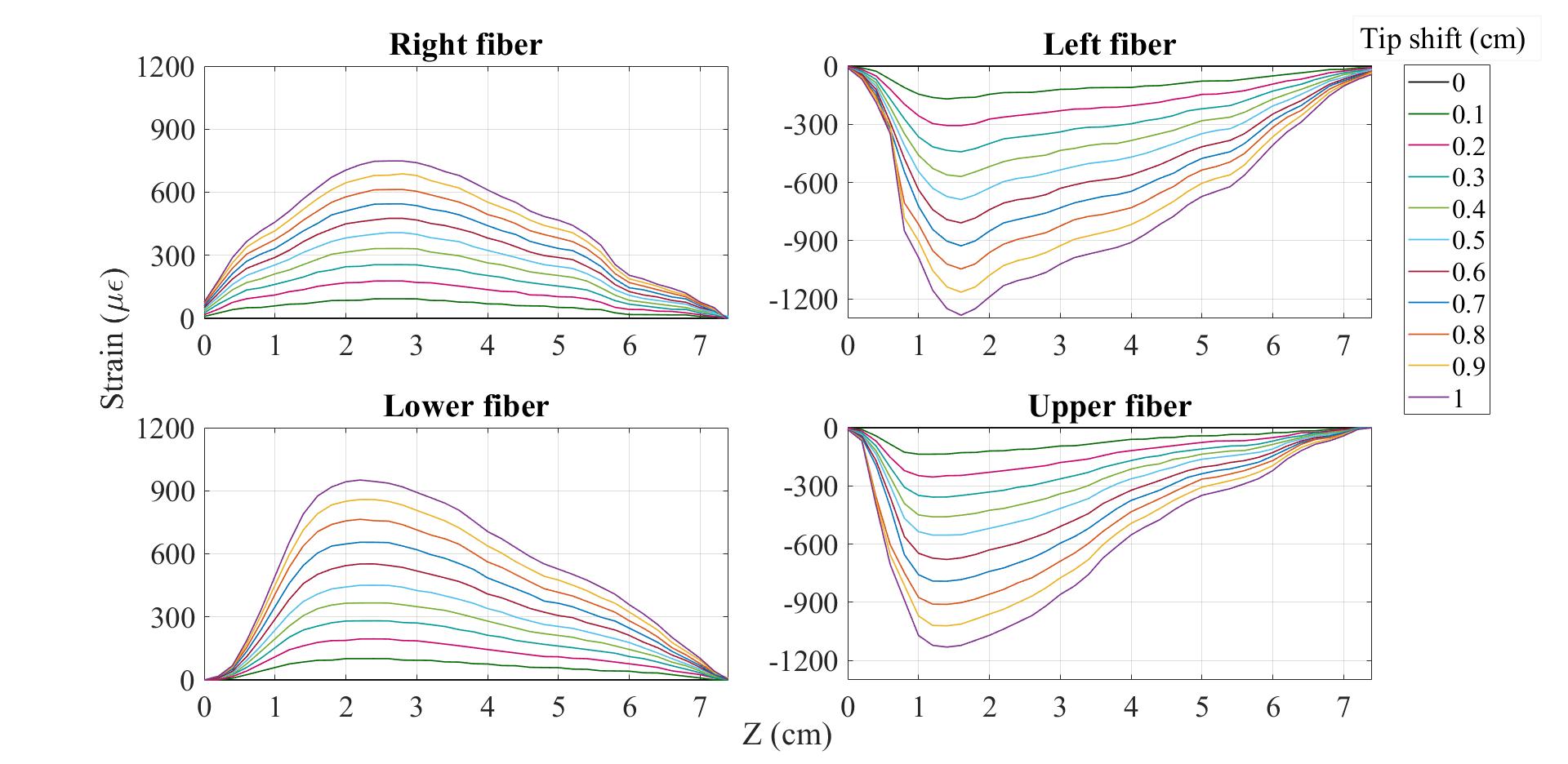


1. Needle bending to the up-left direction.


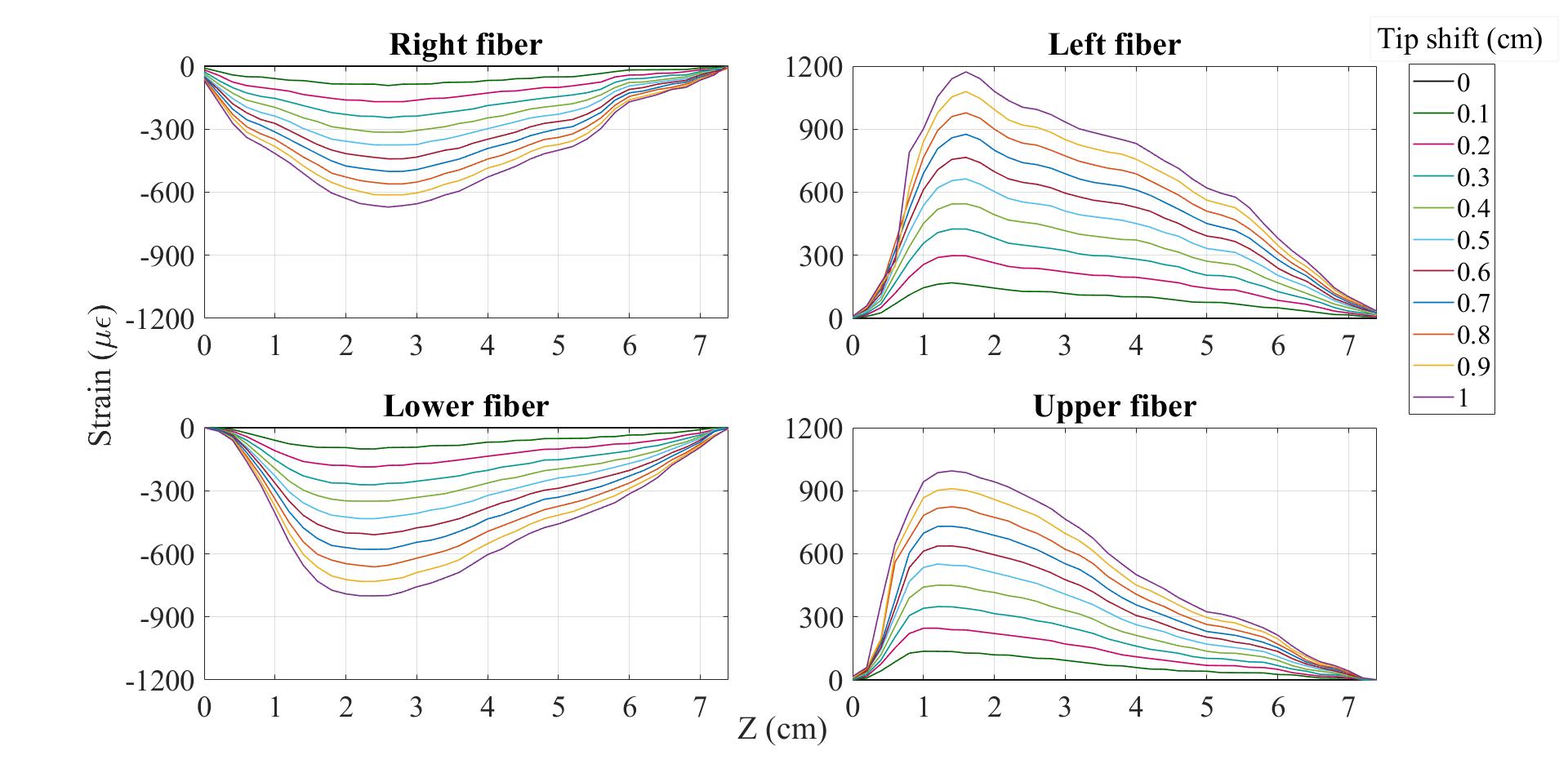


1. Needle bending to the low-right direction

Figure S1. Strain detected by all four fibers over the length of the needle during its bending to three main directions (a-c) and to four intermediate directions (d-g).

Figure S2 illustrates the comparison of the needle shape reconstructed with the algorithm (red) and the reference needle shape (black) during the needle bending to three main directions, such as upper (a), left (b), right (c), and to four intermediate directions, namely low-left (d), up-right (e), up-left (f), low-right (g). As in the case of the inclination to the lower direction (see Fig. 2), the reference is moved from the origin because the first point (z = 0 cm) is located 0.6 cm from the base of the needle and is exposed to displacement with respect to it. Therefore, in the further analysis the reference is moved to the origin in order to find the relative position of the whole needle with respect to this first point. The red line representing the reconstructed shape is inclined to the correct direction but with smaller displacement, which is in agreement with the case of the bending to the lower direction. In order to compensate for this underestimation, the average correction coefficients for left-right and upper-lower fibers have been calcualted based on all trials of the experiment. The shapes comparison after the correction are illustrated in Fig. S3.


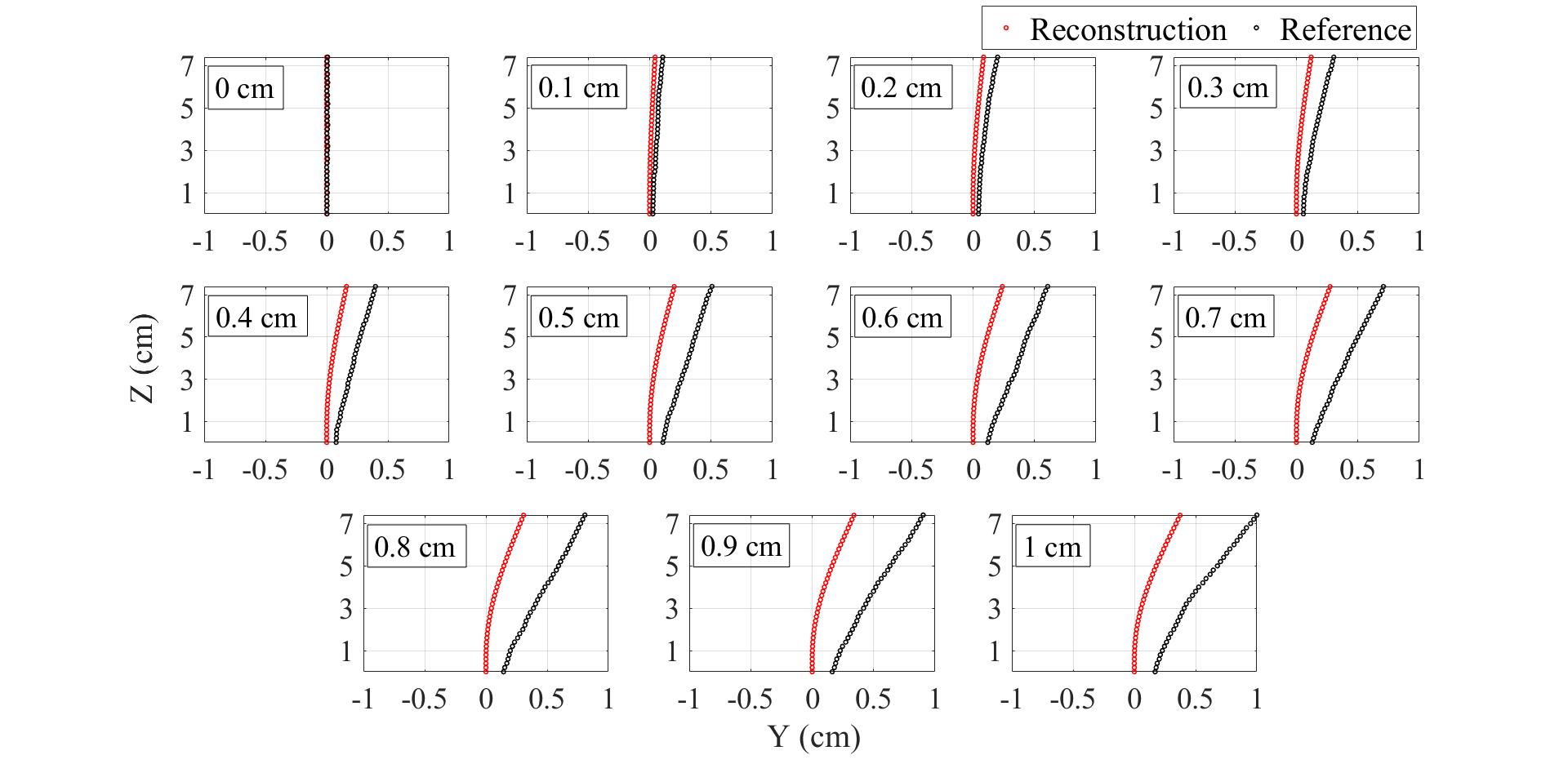


1. Needle bending to the upper direction


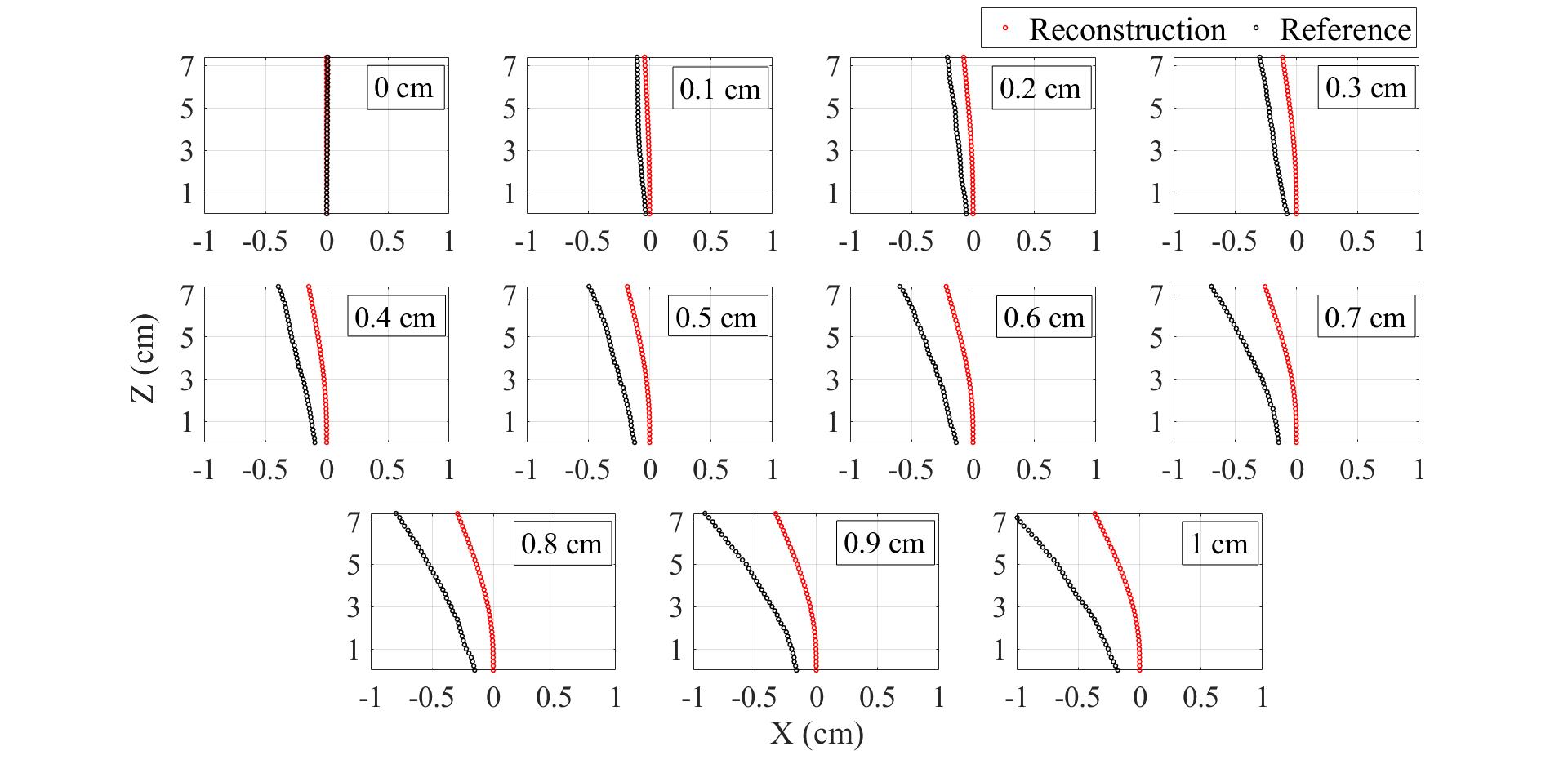


1. Needle bending to the left direction.


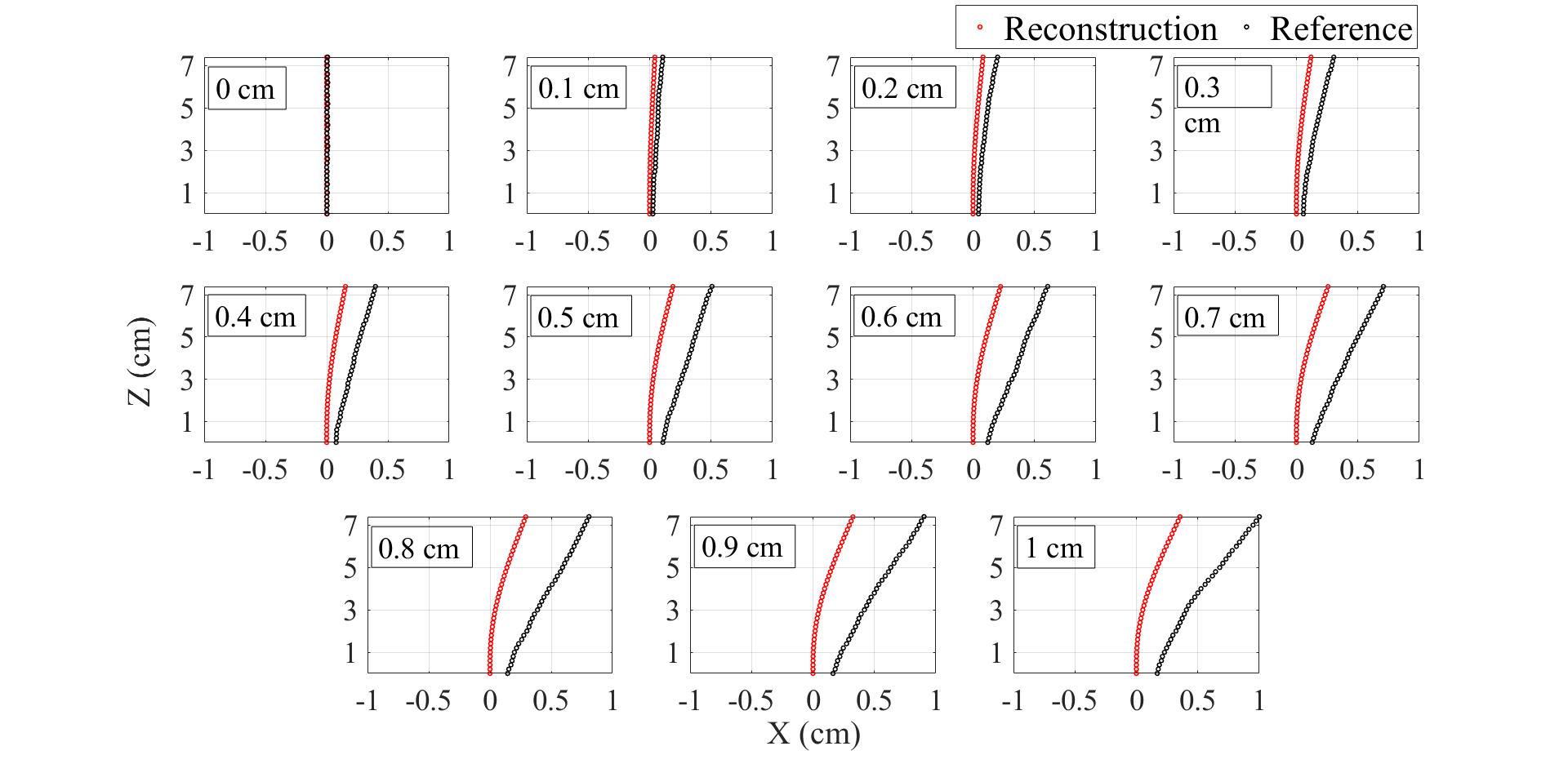


1. Needle bending to the right direction.


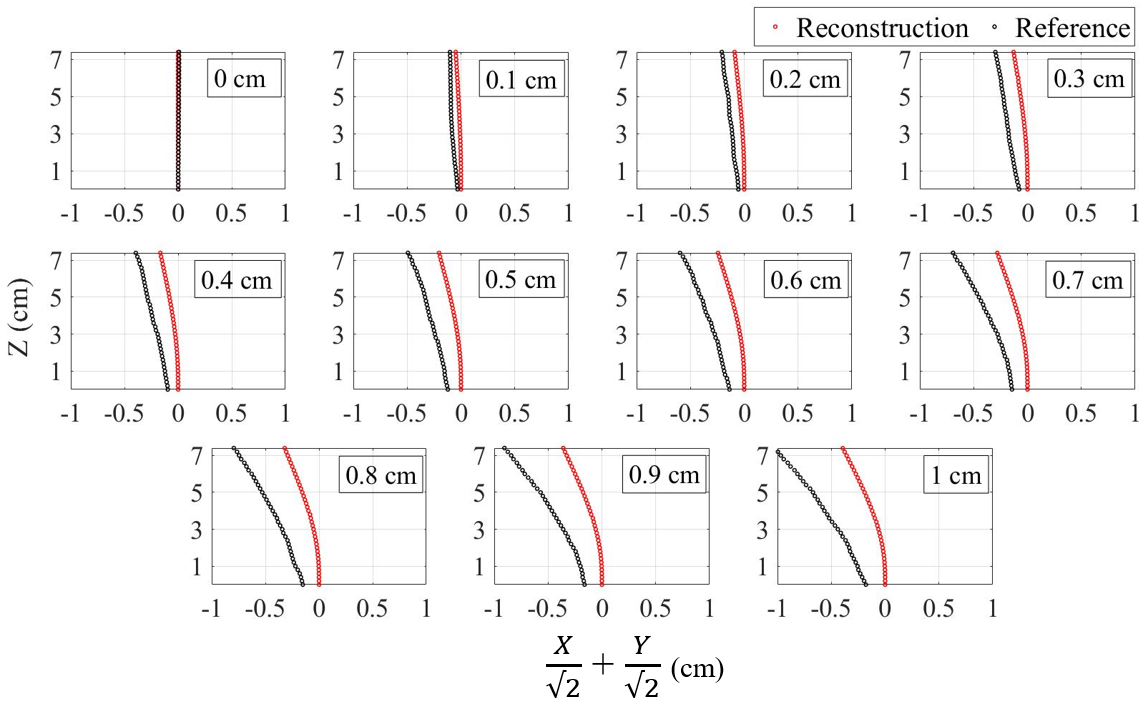


1. Needle bending to the low-left direction.


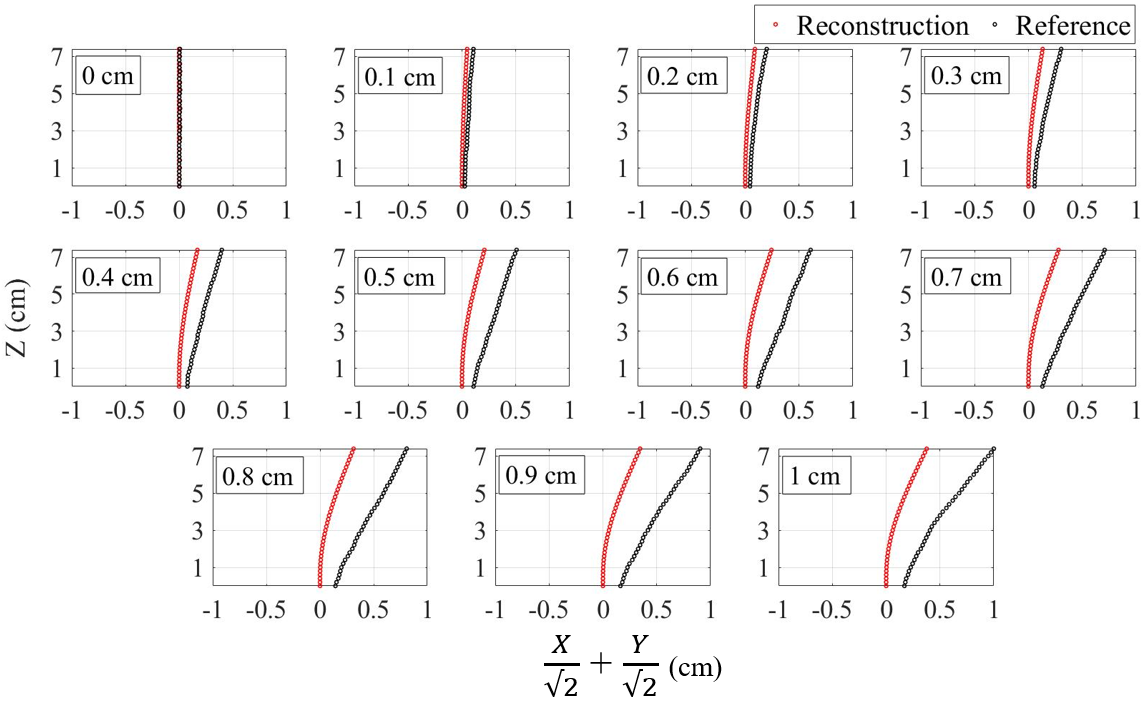


1. Needle bending to the up-right direction.


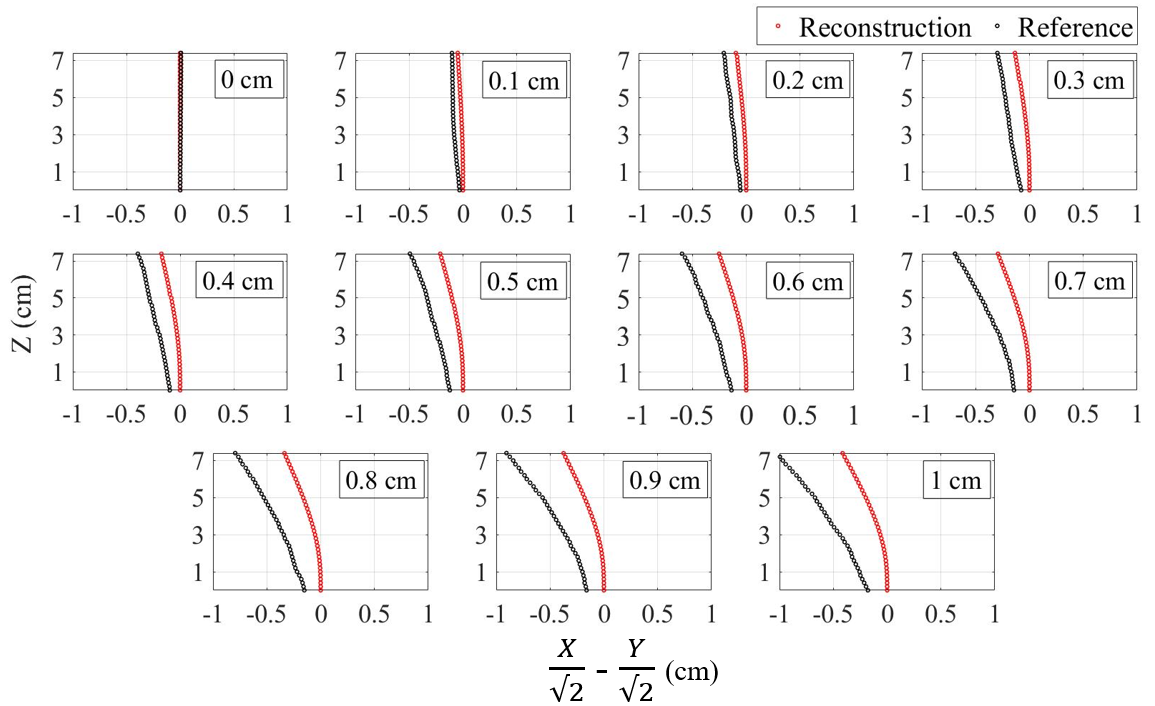


1. Needle bending to the up-left direction.


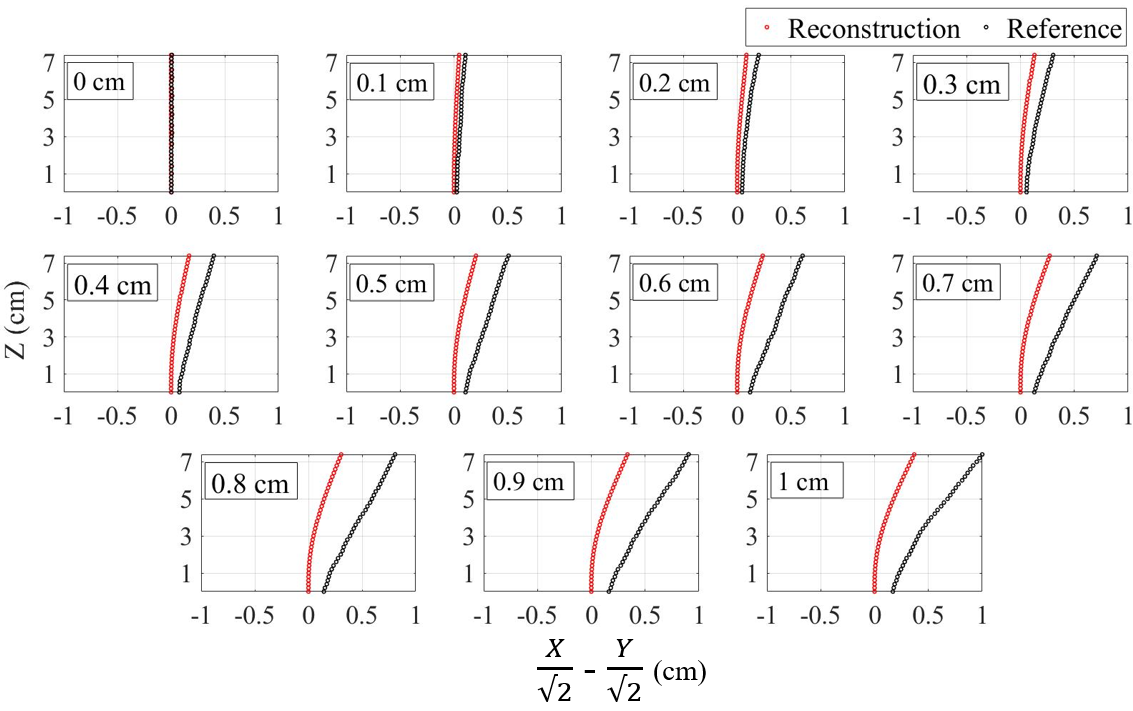


1. Needle bending to the low-right direction.

Figure S2. Comparison of the reconstructed shape (red) with the reference (black) before the application of the correction coefficients during the needle bending to three main directions (a-c) and four intermediate directions (d-g)

Figure S3 illustrates the comparison of the needle shape reconstructed with the algorithm (red) and the reference needle shape (black) after the application of the correction coefficients during the needle bending to three main directions (a-c) and to four intermediate directions (d-g). As can be seen, the reconstructed shape after the correction is closer to the referenced shape than before the correction shown in Fig. S2. The reconstrcution error is still present, but mostly in the middle of the needle, not on its tip. Therefore, the positioning of the tip can be achieved with a high accuracy with the use of this algorithm.


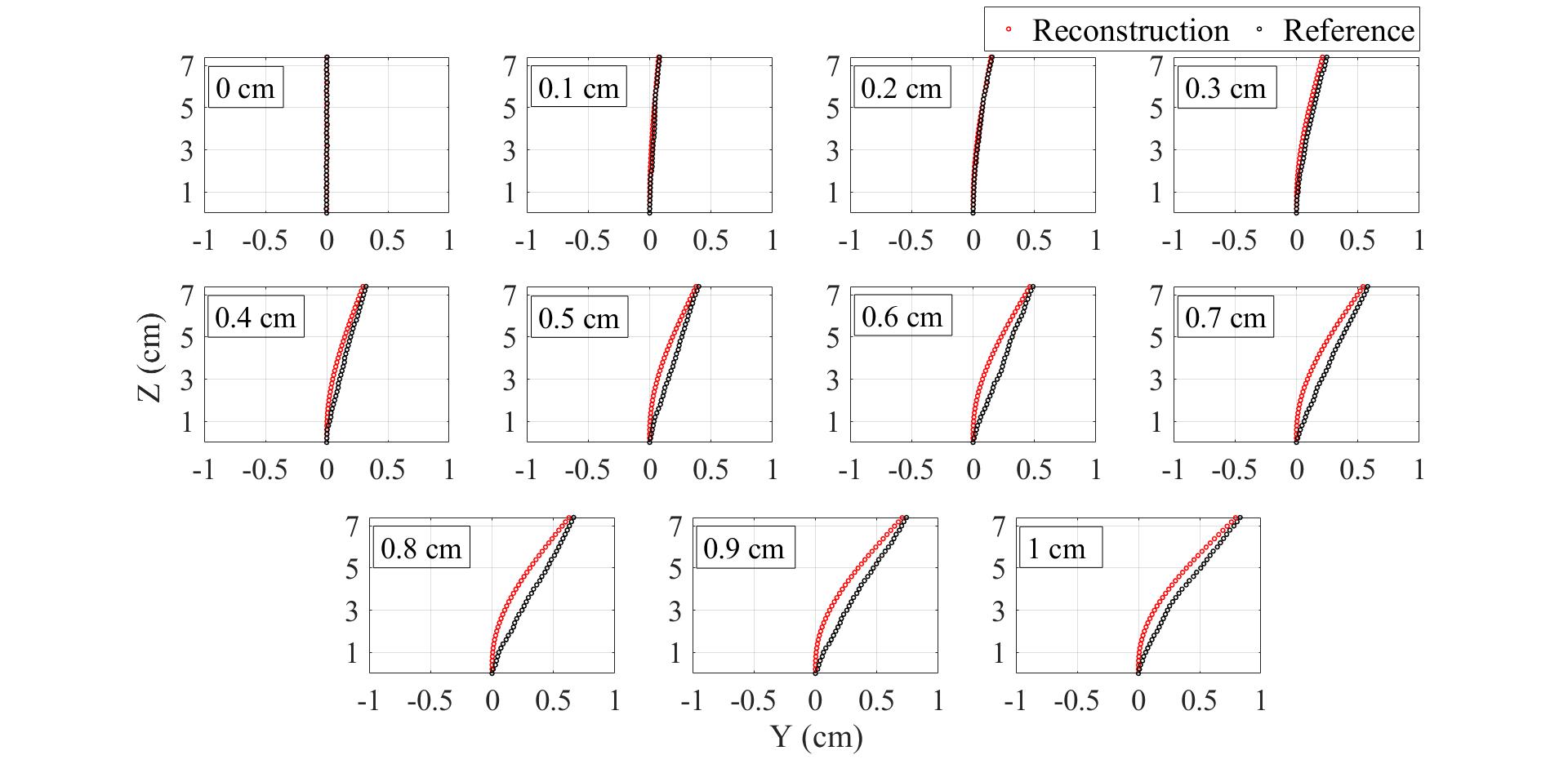


1. Needle bending to the upper direction.


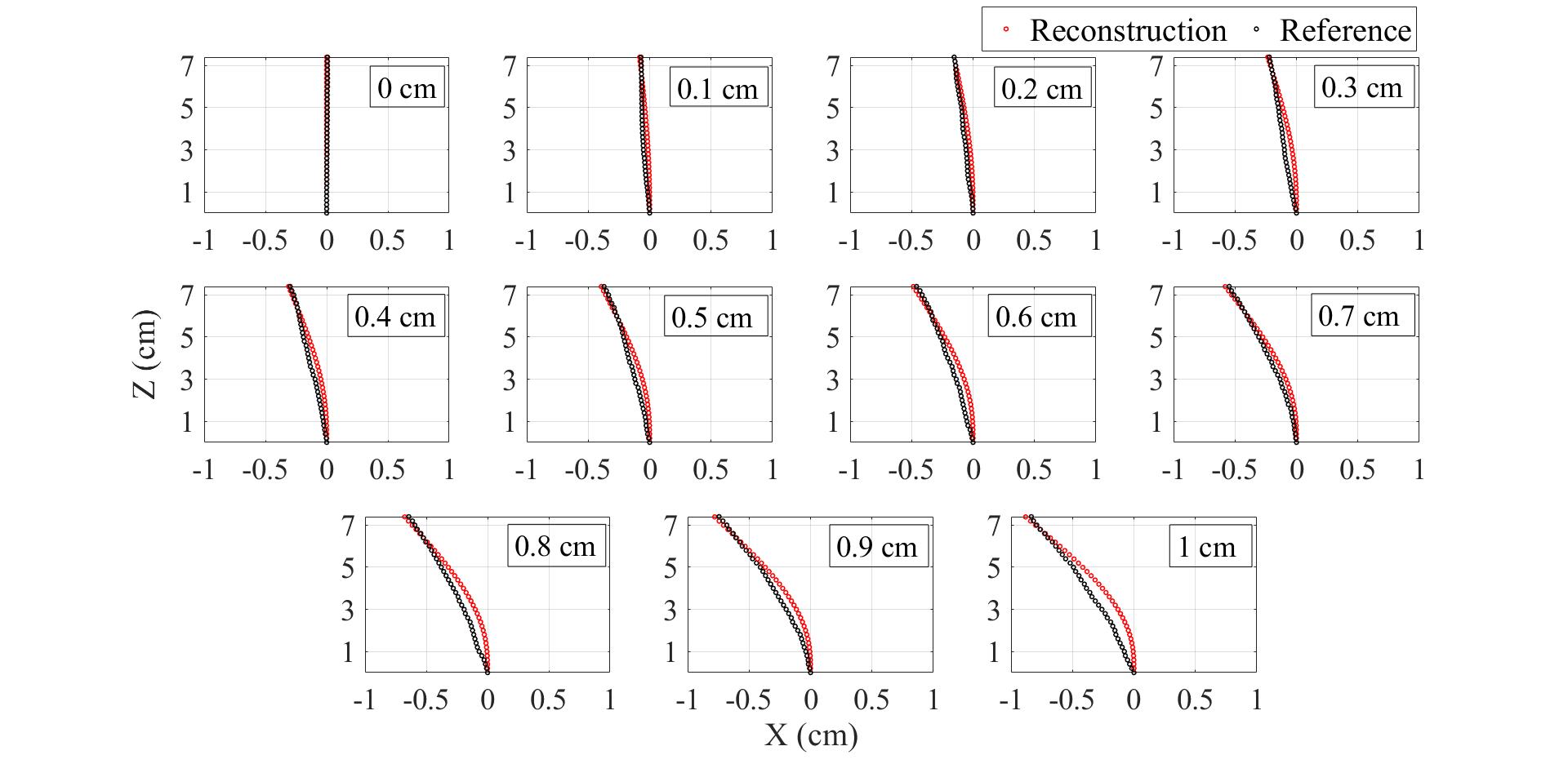


1. Needle bending to the left direction.


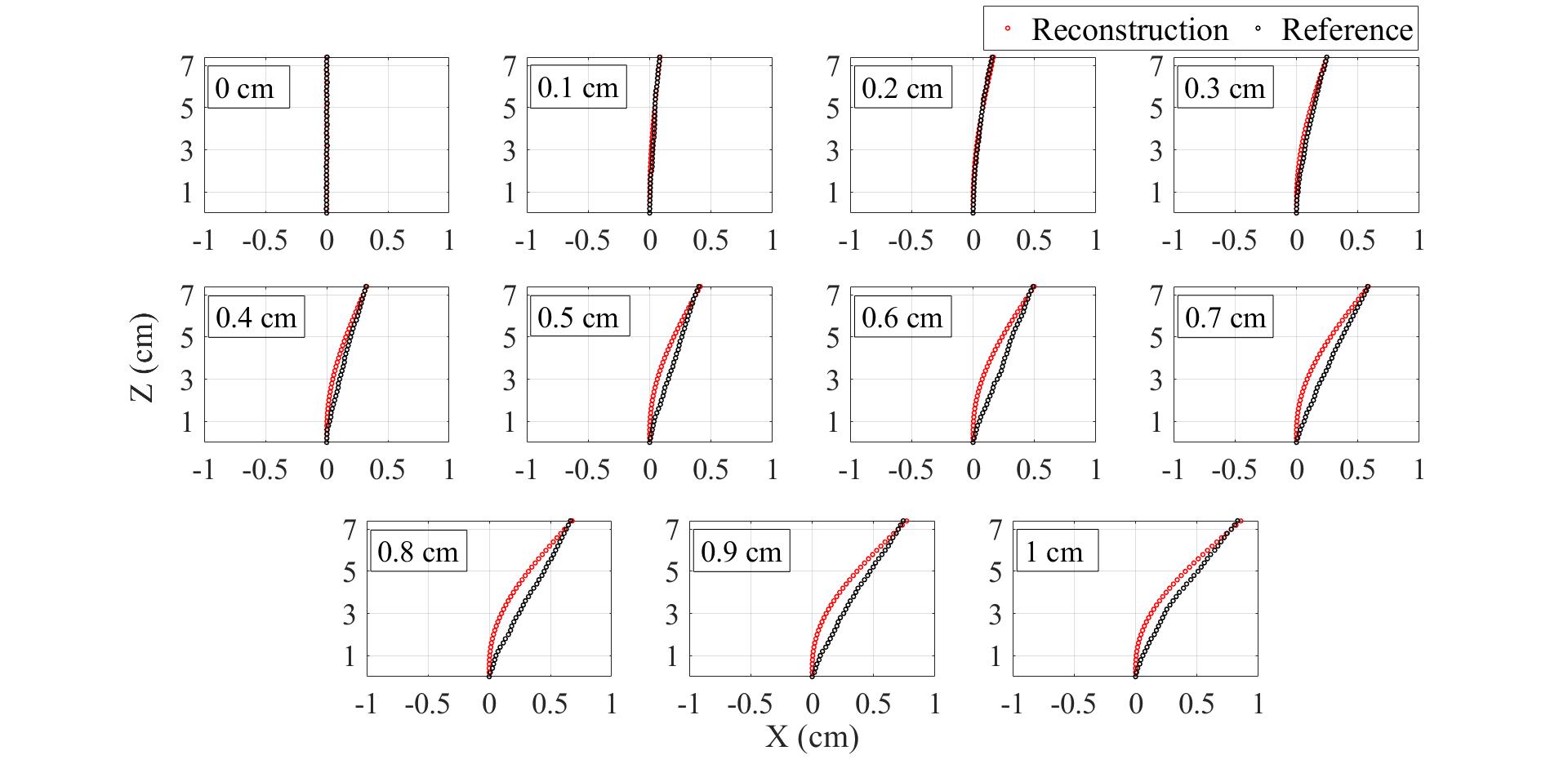


1. Needle bending to the right direction.


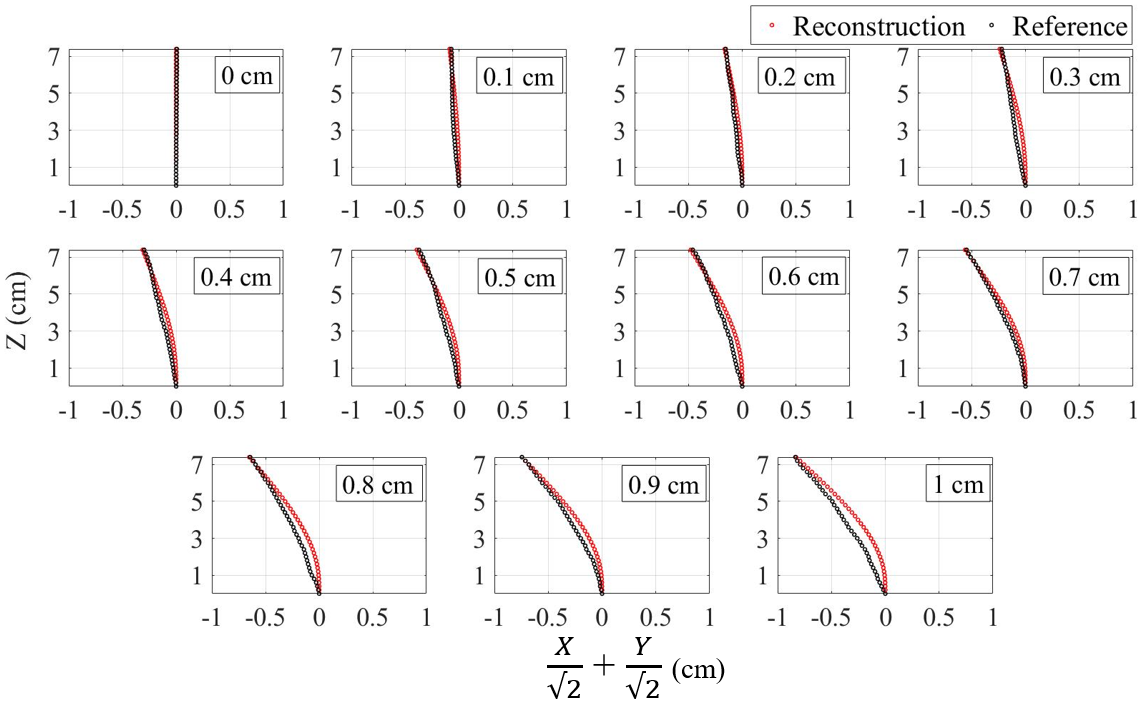


(d) Needle bending to the low-left direction.


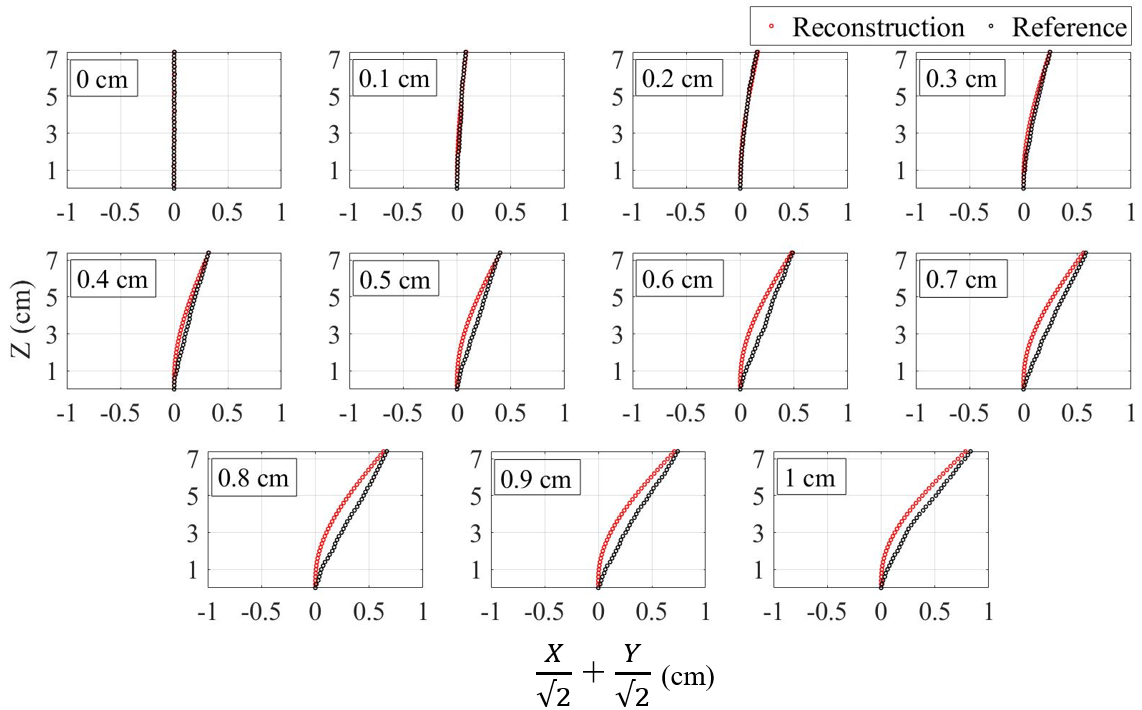


1. Needle bending to the up-right direction.


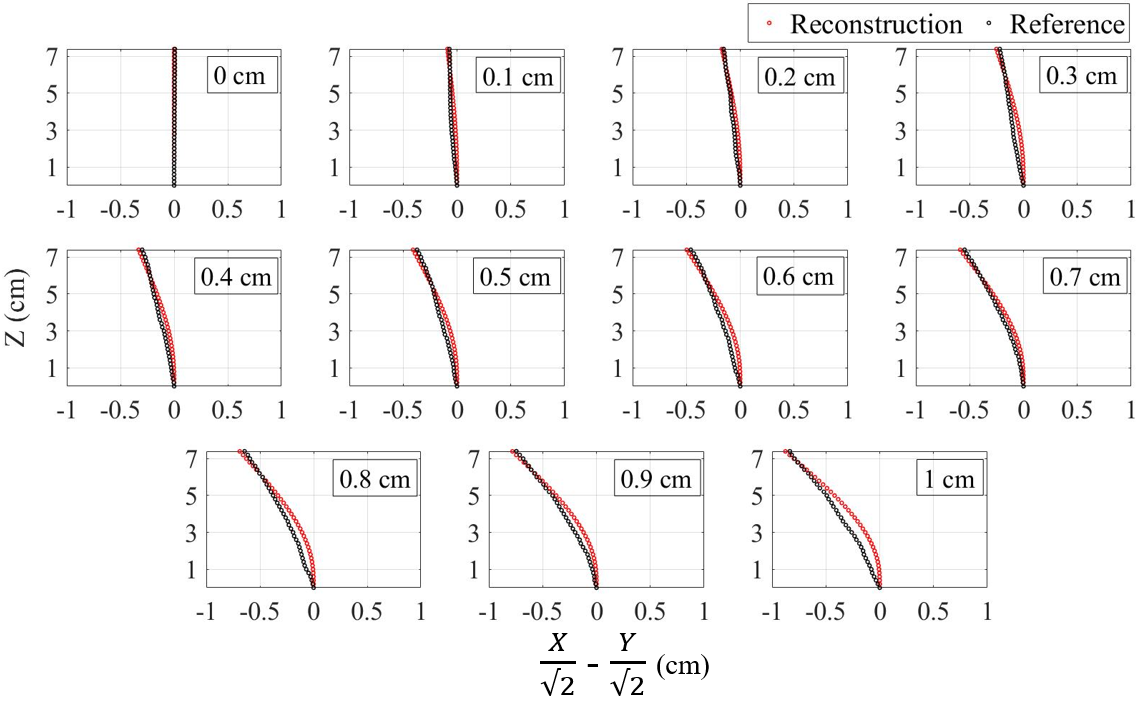


(f) Needle bending to the up-left direction.


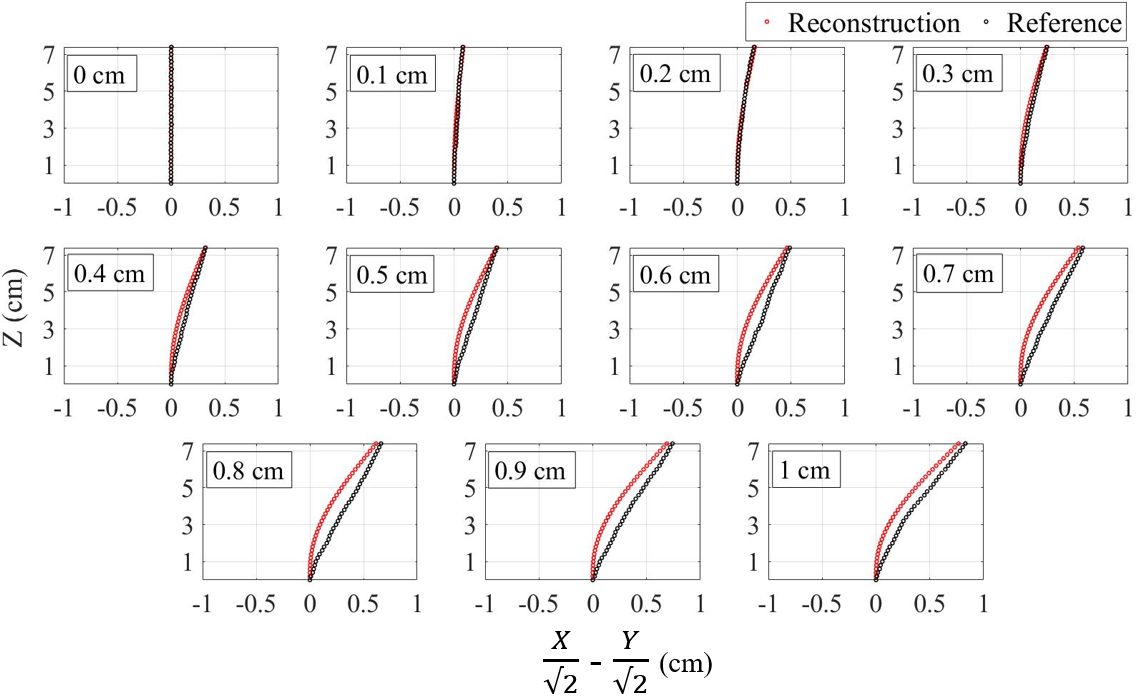


(g) Needle bending to the low-right direction.

Figure S3. Comparison of the reconstructed shape (red) with the reference (black) after the application of the correction coefficients during the needle bending to three main directions (a-c) and four intermediate directions (d-g)
